# Brain growth until adolescence after a neonatal focal injury: sex related differences beyond lesion effect

**DOI:** 10.3389/fnins.2024.1405381

**Published:** 2024-08-23

**Authors:** Pierre-Yves Postic, Yann Leprince, Soraya Brosset, Laure Drutel, Emeline Peyric, Ines Ben Abdallah, Dhaif Bekha, Sara Neumane, Edouard Duchesnay, Mickael Dinomais, Mathilde Chevignard, Lucie Hertz-Pannier

**Affiliations:** ^1^CEA Paris-Saclay, Frederic Joliot Institute, NeuroSpin, UNIACT, Gif-sur-Yvette, France; ^2^INSERM, Université Paris Cité, UMR 1141 NeuroDiderot, InDEV, Paris, France; ^3^Sorbonne Université, CNRS, INSERM, Laboratoire d'Imagerie Biomédicale (LIB), Paris, France; ^4^LP3C, Rennes 2 University, Rennes, France; ^5^French National Reference Center for Pediatric Stroke, CHU de Saint-Etienne, Saint-Etienne, France; ^6^Pediatric Neurology Department, HFME, Hospices Civils de Lyon, Lyon, France; ^7^Université Paris-Saclay, UVSQ – APHP, Pediatric Physical Medicine and Rehabilitation Department, Raymond Poincaré University Hospital, Garches, France; ^8^CEA Paris-Saclay, Frederic Joliot Institute, NeuroSpin, BAOBAB/GAIA/SIGNATURE, Gif-sur-Yvette, France; ^9^Department of Physical Medicine and Rehabilitation, Angers University Hospital Centre, Angers, France; ^10^Rehabilitation Department for Children with Acquired Brain Injury, Saint Maurice Hospitals, Saint Maurice, France; ^11^Sorbonne University, GRC 24 Handicap Moteur Cognitif et Réadaptation (HaMCRe), Paris, France

**Keywords:** brain morphometry, brain growth trajectories, early brain injury, sex effect, multifactorial comparative analysis, male perinatal brain vulnerability

## Abstract

**Introduction:**

Early focal brain injuries lead to long-term disabilities with frequent cognitive impairments, suggesting global dysfunction beyond the lesion. While plasticity of the immature brain promotes better learning, outcome variability across individuals is multifactorial. Males are more vulnerable to early injuries and neurodevelopmental disorders than females, but long-term sex differences in brain growth after an early focal lesion have not been described yet. With this MRI longitudinal morphometry study of brain development after a Neonatal Arterial Ischemic Stroke (NAIS), we searched for differences between males and females in the trajectories of ipsi- and contralesional gray matter growth in childhood and adolescence, while accounting for lesion characteristics.

**Methods:**

We relied on a longitudinal cohort (AVCnn) of patients with unilateral NAIS who underwent clinical and MRI assessments at ages 7 and 16 were compared to age-matched controls. Non-lesioned volumes of gray matter (hemispheres, lobes, regions, deep structures, cerebellum) were extracted from segmented T1 MRI images at 7 (Patients: 23 M, 16 F; Controls: 17 M, 18 F) and 16 (Patients: 18 M, 11 F; Controls: 16 M, 15 F). These volumes were analyzed using a Linear Mixed Model accounting for age, sex, and lesion characteristics.

**Results:**

Whole hemisphere volumes were reduced at both ages in patients compared to controls (gray matter volume: −16% in males, −10% in females). In ipsilesional hemisphere, cortical gray matter and thalamic volume losses (average −13%) mostly depended on lesion severity, suggesting diaschisis, with minimal effect of patient sex. In the contralesional hemisphere however, we consistently found sex differences in gray matter volumes, as only male volumes were smaller than in male controls (average −7.5%), mostly in territories mirroring the contralateral lesion. Females did not significantly deviate from the typical trajectories of female controls. Similar sex differences were found in both cerebellar hemispheres.

**Discussion:**

These results suggest sex-dependent growth trajectories after an early brain lesion with a contralesional growth deficit in males only. The similarity of patterns at ages 7 and 16 suggests that puberty has little effect on these trajectories, and that most of the deviation in males occurs in early childhood, in line with the well-described perinatal vulnerability of the male brain, and with no compensation thereafter.

## 1 Introduction

Plasticity stands as an intrinsic property of the central nervous system, reflecting its inherent capability to dynamically respond to the environment and experiences. In the developing brain, the high level of plasticity is generally considered beneficial, but the specific advantages it confers in situations of early brain lesions remain unclear. Patients with early onset focal lesion tend to better recover impaired function at a cost of a broader impairment, suggesting dysconnectivity beyond the lesioned territory, with expected deviations in global and regional brain growth. The influence of clinical factors such as lesion characteristics and epilepsy have been explored (Sullivan et al., 2023), but other factors such as sex, environmental conditions, and intervention/rehabilitation further contribute to the complexity of understanding neural plasticity (Anderson et al., 2014). In particular, the exquisite vulnerability of male brains in the perinatal and early childhood period has been long suspected, considering the sex ratio of a number of early-onset neurodevelopmental disorders such as autism spectrum disorders (ASD) (Halladay et al., 2015), attention-deficit hyperactivity disorder (ADHD) (Ramtekkar et al., 2010), obsessive compulsive disorder (OCD) (Mathes et al., 2019). Several studies also reported a male bias in prevalence of cerebral palsy with a male/female ratio of 1.4/1 (Stanley et al., 2000; Smithers-Sheedy et al., 2014). However, long term sex-related differences in clinical outcome remain debated (Romeo et al., 2011, 2023)

Neonatal arterial ischemic stroke (NAIS) (Fluss et al., 2019; Giraud et al., 2023) is a valuable model for examining brain plasticity after an early brain lesion. Although most NAIS are considered to be idiopathic, several studies describe potential risk factors (Li et al., 2017). Notably, males are disproportionately affected by NAIS (2/3 of the NAIS population) (Dunbar and Kirton, 2018; Dunbar et al., 2020), suggesting a sex-related risk factor. Though children with NAIS form a highly homogeneous population with regard to lesion onset and localization [Middle Cerebral Artery (MCA) territory in 85% of cases, in the left hemisphere in 2/3 of cases] (Kirton and deVeber, 2013), they exhibit a broad spectrum of long term outcome (Chabrier et al., 2011; Dunbar and Kirton, 2018).

Morphometric analyses, focusing on brain growth as a first approximation of early brain plasticity through volumes changes, have been conducted on subjects after perinatal stroke (Dunbar and Kirton, 2019). A recent study demonstrated generalized lower cortical gray matter volume, thickness, and a few gyrification differences in the contralesional hemisphere compared to an age-corrected control group (Shinde et al., 2023). Other studies have highlighted lower white matter volumes along fibers connected to the lesion (Moses, 2000; Stiles et al., 2003), bilateral thalamus volume alterations (Craig et al., 2019a), bilateral basal ganglia atrophy (Hassett et al., 2022), crossed cerebellar atrophy (Craig et al., 2019b). Bilateral thalamus and basal ganglia atrophy correlated with epilepsy risks (Vaher et al., 2023), as well ipsilesionnal atrophy correlated with poorer hand functions (Ilves et al., 2022) and bilateral hippocampal atrophy correlated with memory impairment (Gold and Trauner, 2014). To our knowledge, only a few studies delved into the brain morphometry of NAIS specifically. One showed volume alteration in cortico-spinal tract in centrum semiovale white matter, and proposed it as a marker of cerebral palsy (Dinomais et al., 2015). Another correlated poorer contralateral hand function with smaller volumes in ipsilesional mediodorsal thalamus, ipsilesional corticospinal tract (superior corona radiata), posterior limb of the internal capsule, the cerebral peduncle and the ipsilesional body of corpus callosum (Dinomais et al., 2016). The last explored thickness, volume, and gyrification alterations in both hemisphere (Al Harrach et al., 2019). It identified localized modifications in cortical areas distant from the primary lesion within the ipsilesional hemisphere, as well as in the contralesional hemisphere, emphasizing subtle differences in patterns between right and left-lesioned patients. In these studies, sex-related differences were not specifically investigated, as in most analyses, sex was introduced as a co-variable in the models, that also accounted for a fairly large variability in ages and clinical factors (age at lesion occurrence, side and extent of the lesion, epilepsy, cerebral palsy, etc…). Most of the previously cited studies rely on cohort with small cohorts with wide age range and different lesion territories and origins.

The goal of the present study was to investigate the contribution influence of sex among the main demographic and clinical co-variables on the longitudinal term global and regional brain growth after a unilateral NAIS, taking advantage of the exceptional resources of our prospective longitudinal French AVCnn cohort (Dinomais et al., 2015; Chabrier et al., 2016), and using i. Very strict inclusion criteria based on the combination of clinical and imaging data (only unilateral NAIS). ii. A long term follow up with a complete clinical and MRI research protocol at 2 key childhood ages: around 7 year old (7 yo, beginning of elementary school) and 16 year old (16 yo, before professional orientation); iii. A study design enabling a systematic and consistent longitudinal assessment (i.e., comparability between ages of clinical and behavioral tests, monocentric 3T MR evaluation with imaging parameters matched at both ages and an image analysis pipeline dedicated to longitudinal comparisons). By measuring hemispheric [cortical gray matter (GM) and white matter (WM)] volumes, as well as regional cortical and sub-cortical GM volumes, we aimed to assess: (1) the brain growth trajectories of the patients between 7 and 16 years of age, in both sexes, compared to the control groups; (2) the impact of the studied variables on growth patterns; and (3) the differences in patients volumes according to sex and age (pre-pubertal period and puberty effect). We expected global and focal growth deficits in patients, mostly in ipsilesional areas, but also within the contralesional hemisphere, more pronounced in males compared to females, and possibly evolving between 7 and 16 years of age due to puberty.

## 2 Material and methods

### 2.1 Participants

#### 2.1.1 Patients

Patients were part of the AVCnn cohort (Accident Vasculaire Cérébral du nouveau-né) (PHRC régional n80308052, and PHRC interrégional n81008026—eudract number 2010-A00329-30) (Chabrier et al., 2016). Initially, the cohort comprised 100 term neonates with a NAIS, confirmed by imaging in the acute phase. These infants were consecutively enrolled between November 2003 and October 2006 from 39 neonatology and neuropediatric units throughout mainland France.

At the age of 7–8 years (2010– 2013), a research protocol involving neuropsychological and MRI investigations was proposed to the 80 children still in active file (7yo session). The clinical description of the 7-year-old cohort (*n* = 73) has been published in Chabrier et al. (2016). In summary, males represented two-thirds of the patients, lesions were located in the left hemisphere in two-thirds of cases, and 85% involved the MCA territory. Of these, 52 [35 males (M), 17 females (F)] adhered to the imaging protocol.

Subsequently, at the age of 16–18 years, all AVCnn patients still in active file (*n* = 50) were contacted again to participate in the AVCnnADO follow-up research project (16 yo session) (ethical approval ID-RCB 2020-100106-33). They were offered a comprehensive two-day session with clinical, language, and neuropsychological assessments, as well as MR imaging over 2 days. All responders (*n* = 32) but one had participated in the 7 yo session. One patient not belonging to the original cohort (thus not having done the 7 yo session), was recruited, as this patient fulfilled all strict criteria (in terms of age and NAIS characterization according to her clinical reports and imaging in the neonatal period). Eventually, 33 patients (13 females, 20 males) participated in both the clinical assessments and the imaging protocol, after obtaining parent consents and adolescent assents.

To ensure patients homogeneity, precise lesion characterization (territory and extent) was obtained for each patient through consensus within an expert panel of pediatric neuroradiologists, neurologists, and rehabilitation physicians. Lesion territories were defined as uni or bilateral, in Anterior Cerebral Artery (ACA), Middle Cerebral Artery (MCA), and Posterior Cerebral Artery (PCA), based on scar tissue sequelae (including tissue loss on T1w images and gliosis on FLAIR). The MCA territory was further subdivided into: i. frontal (inferior or middle frontal gyrus, frontal operculum of the insula, precentral gyrus); ii. temporal (middle and posterior parts of the superior temporal gyrus); iii. parietal (postcentral gyrus, supramarginal gyrus, intraparietal sulcus, superior parietal lobule, angular gyrus, and parietal operculum of the insula); iv. basal ganglia; and v. the entire MCA territory. A combination of several territories within the MCA territory was frequent. This *post-hoc* review led to the exclusion of 7 cases at 7 yo and 3 at 16 yo that did not fully match unilateral arterial ischemic stroke definition, mostly corresponding to bilateral junctional ischemia due to hypoxic-ischemic encephalopathy. The very few patients with PCA stroke (*N* = 2 at 7 yo, 1 at 16 yo) were excluded from this study. NAIS in MCA and ACA territories accounted, respectively, for 93 and 7% of 7 yo cases, and 90 and 10% of 16 yo cases.

To address the variability in lesion size and severity, ranging from focal cortical ribbon interruption to strokes involving the entire MCA territory including basal ganglia, a regressor of lesion severity was deemed essential for statistical analyses. However, using lesion volumes at ages 7 or 16 was unsuitable for this purpose. Indeed, the residual cavities at 7 or 16 years poorly reflected the size of the initial stroke documented from the neonatal period due to tissue destruction, retraction, and brain growth since birth. For example, some patients missing one or more entire gyrus showed no or very limited apparent cavity, with very limited gliosis which did not reflect the extent of the initial lesion either. Obtaining lesion volume at birth was not feasible for all subjects due to inadequate neonatal MRI data collected in primary hospitals and/or the lack of digital data. We thus introduced a lesion severity score, which combined cortical and sub-cortical GM structures, based on visual assessment of involved (missing) brain tissue. This score, assessed by an expert neuroradiologist (LHP), was constructed by adding two subscores: 1. lesion affecting sub-cortical GM structures (0 = no; 1 = yes); and 2. extent of lesioned cortical territories (0 = none; 1 = one gyrus; 2 = multiple gyri; 3 = whole arterial territory), inspired by the usual clinical classification of the affected territories in ischemic strokes (superficial, deep, mixed). Building this score and including it as a correcting variable in the analysis (see Section 2.7) allows to correct for both inter- and intra-group lesion severity variability.

Active epilepsy (requiring chronic medication) at 16 y was rare (*n* = 5, 1 F and 4 M), and not severe (no or a few seizures per year under medication). We found no differences of socio-economic status (SES) between males and females, neither at 7 yo (F: 36.9, SD = 10.9; M: 38.4, SD = 14.1) nor at 16 yo (F: 38.3, SD = 14.2; M: 41.8, SD = 15.3, Kolmogorov-Smirnov on SES computed with Hollingshead method). No SES difference was found between patients who came back at 16 yo (longitudinal) and patients who did not (7 yo only: 35.1, SD = 14.7; longitudinal: 37.4, SD = 13.0). No differences in IQ were found between patients who came back at 16 yo (longitudinal) and patients who did not. Unilateral Cerebral Palsy (uCP) was found in 39.5% of patients, in line with previous series, with no clear changes between 7 and 16 years (37.9%), and no significant differences between males and females (Chi-Square). All uCP children could walk, mostly with mild restrictions. Hand dysfunction was more pronounced than foot, with nearly non-functional assisting hand in two patients. The clinical presentation of the analyzed population is summarized in Table 1.

**Table 1:** Clinical presentation of the cohort before imaging quality check.

	**Session 7 yo**	**Session 16 yo**	**Seizure**	**CP**	**GMFCS**	**BFMF**	**SES**
Male	27	17	11.11%	42.86%	1.25	0.86	38.55
					SD = 0.58	SD = 1.01	SD = 13.86
Female	16	12	14.29%	35.29%	1.24	0.52	36.94
					SD = 0.66	SD = 0.72	SD = 10.54

Seizure: active (treated) or past seizure episodes; CP, cerebral palsy; GMFCS, gross motor function classification system, 84% of the subjects had a score of 1; BFMF, bimanual fine motor function, SES, socio-economic score, computed using Hollingshead method.

#### 2.1.2 Healthy controls

During the 7 yo session, a total of 37 age-matched controls (19 F, 18 M) were enlisted. These controls underwent a succinct standard clinical screening encompassing personal and family history, schooling, and a brief clinical examination. Then, they underwent an MRI study identical to that administered to the patients.

In the 16 yo session, 31 age-matched controls (16 F, 15 M) were included for comparative analysis. Similar to the earlier session, these controls underwent succinct standard clinical screening, including personal and family history, schooling, and a brief clinical examination. Furthermore, participants were underwent the Raven matrices subtest of the Weschler Adult Intelligence Scale (WAIS-IV) to screen for any conspicuous deviations from typical cognitive development. Lastly, they underwent the complete MRI study, similarly to the protocol applied to the patients.

Eventually, among all groups, 27 patients (11 F, 16 M) and 17 controls (8 F, 9 M) underwent assessments at both ages, providing unique long term longitudinal data.

In this study, the “sex” variable refers to the sex assignment made at birth by professionals and parents, and subsequently self-reported by the children then adolescents. No disparities between sex assignment and self-reported sex were identified along the study.

### 2.2 MRI acquisition

For this morphometric study, 3D T1-weighted images were acquired at both ages as part of a multimodal 3T MRI acquisition including diffusion MRI, motor and language functional MRI and resting state fMRI, in addition to FLAIR images to detect gliosis and scarring, in Neurospin, CEA-Saclay, France.

Considering the long delay between the sessions, the 3T MR scanner underwent a complete upgrade between the first and second session from a Siemens MAGNETOM Trio Tim system, with a 12-channel head coil to a Siemens MAGNETOM Prisma fit system, with a 64 channel head coil. To optimize image comparability between the two sessions, we selected MPRAGE sequence parameters to provide a similar contrast as follows: Session 7 yo, TR = 2,300 ms, TE = 4.18 ms, TI = 900 ms, voxel size 1 × 1 × 1 mm^3^, 176 slices. Session 16 yo, TR = 2,300 ms, TE = 3.05 ms, TI = 900 ms, voxel size = 0.9 × 0.9 × 0.9 mm^3^, 176 slices.

T2 FLAIR images were acquired for lesion delineation with the following parameters: At Session 7 yo, TR = 5,000 ms, TE = 395 m, voxel size 0.9 × 0.9 × 1 mm^3^, 160 slices. At Session 16 yo, TR = 5,000 ms, TE = 396 ms, voxel size= 0.9 × 0.9 × 0.9 mm^3^, 176 slices.

### 2.3 Image processing

The methodology presented below has been designed to enable accurate image processing in the context of a developmental study and in the presence of brain lesions, and to ensure good performance across the whole cohort.

T1-weighted images were processed as shown in Figure 2. Raw DICOM images were imported, anonymized, according to the BIDS specification (Gorgolewski et al., 2016). Brain masks and extracted brain images (“skull stripping”) were generated using HD-BET (Isensee et al., 2019). A detailed Quality check was made along the process, first visually to ensure correct registration, GM/WM segmentation and ROI delineation. Raw T1 images with quality issues (mostly due to motion artifacts) were evaluated by combining Image Quality Metrics obtained with MRIQC (Esteban et al., 2017), and the Euler Number (Rosen et al., 2018)and CAT12 Image Quality Rating (IQR) (Gaser et al., 2024). By thresholding the IQR vs. Euler Number distribution, compared to visual assessments, seven images of insufficient quality at 7 yo were excluded. Post-quality check dataset comprised 37 patients at 7 yo (22 M, 15 F; 94.6% MCA, 6.4% ACA) and 29 images at 16 yo (17 M, 12 F; 90% MCA, 10% ACA).

### 2.4 Lesion mapping

Lesion masks were manually drawn at 7 yo, by two expert neuroradiologists using ITK-SNAP on both T1-weighted images (tissue destruction and porencephaly) and T2-FLAIR images (gliosis). Each final mask resulted from the fusion of both T1w and T2-FLAIR masks, followed by a morphological closing process. Discrepancies between experts were solved through consensus.

At 16 yo, lesion masks were computed from the 7 yo masks through intra-subject registration (ANTs) (Avants et al., 2008) of the T1w images between the ages of 7 and 16. This intra-subject registration used HD-BET brain masks at both 7 and 16 years as complementary inputs (registration masks). In the few instances where 16-year-old subjects lacked usable anatomical images at 7 yo, the masks were drawn using the same manual method.

**Figure 1:**
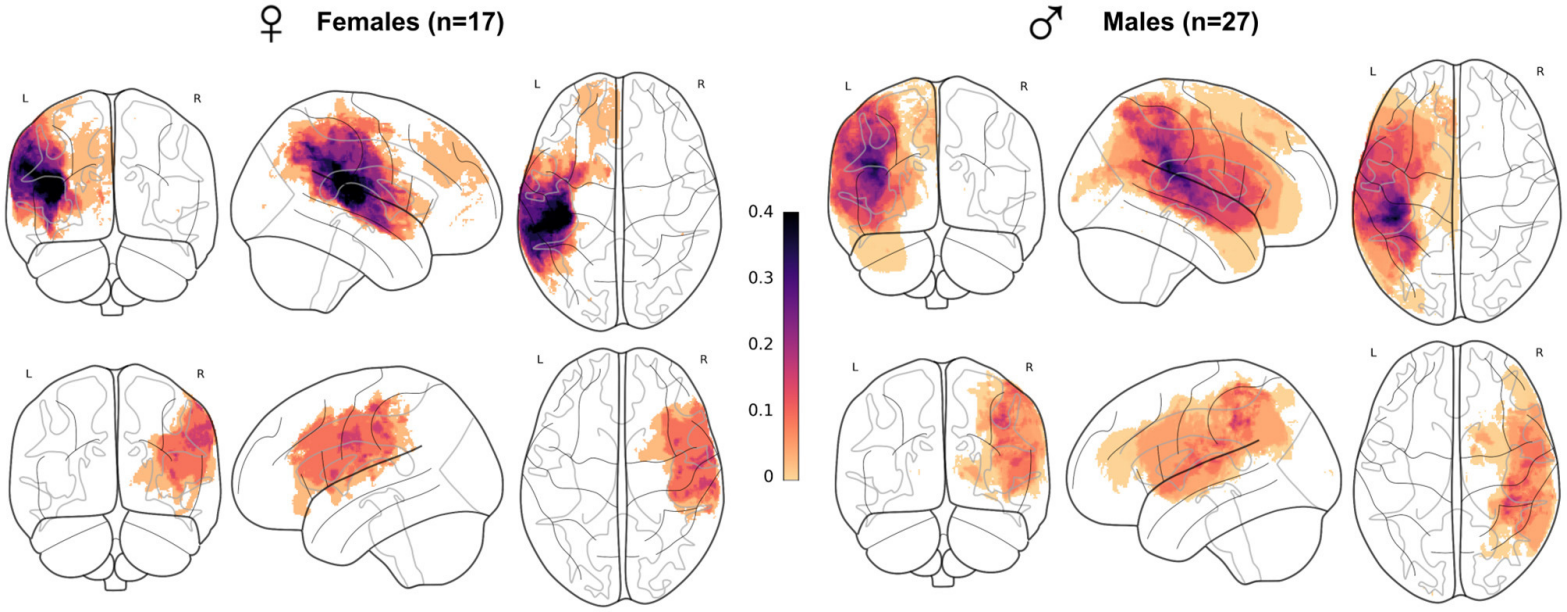
Lesion mapping per sex subgroup in the whole patient cohort. Superposition of all patient's lesion masks per sex and lesion side (females lesions represented on the left-hand side) and lesion side (left hemisphere lesions on the first row), visualized as a maximum intensity projection. Each subject, longitudinal or not, was counted only once. All masks were registered in MNI ICBM152 2009c symmetric template. Mask count per voxel was divided by the total size of sex subgroups.

**Figure 2:**
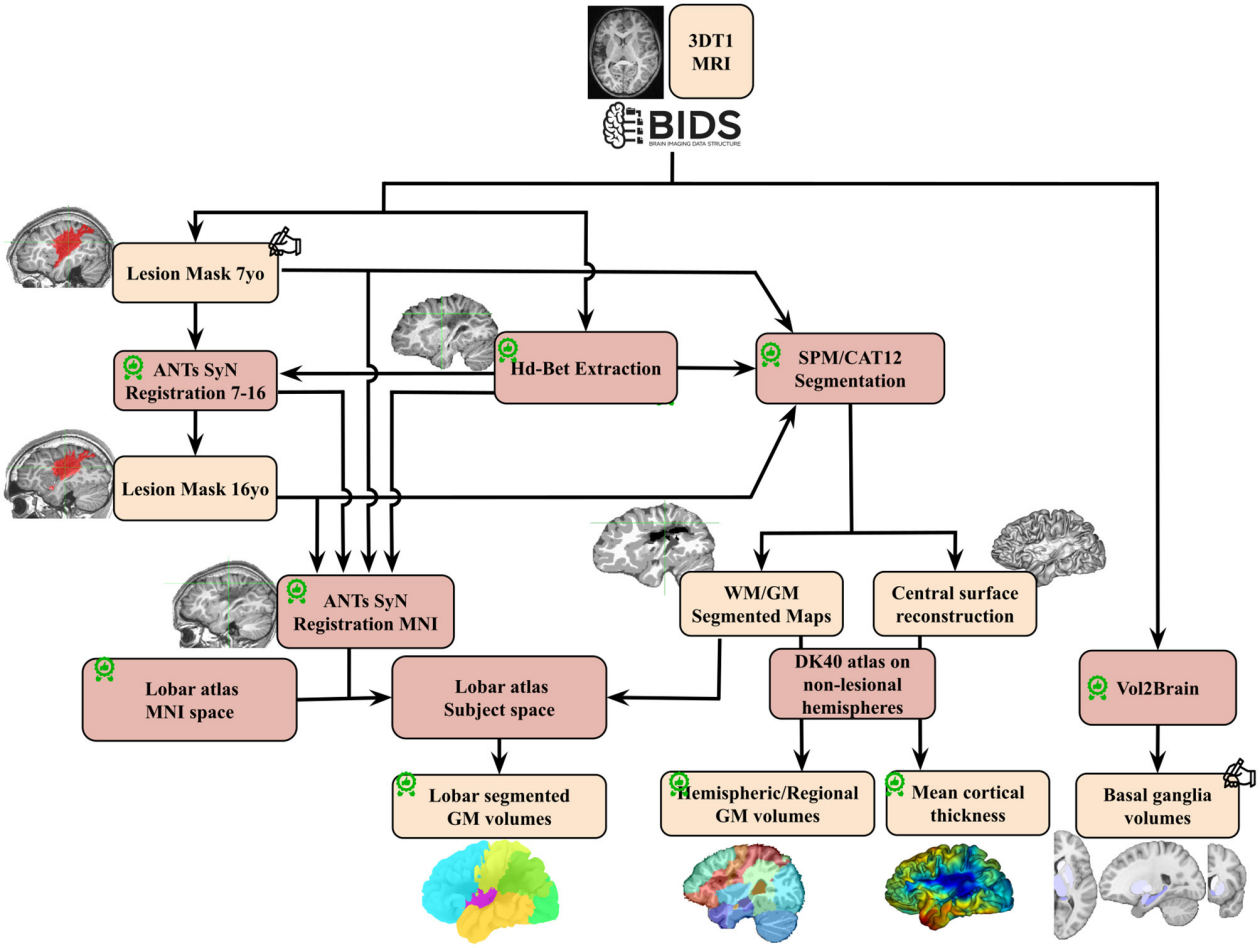
Image processing pipeline performed on T1 images for the whole cohort. 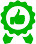: quality check; 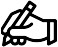: made/corrected by trained radiologist; GM, gray matter; WM, white matter; DK 40, Desikan Atlas.

Lesion mapping (overlap of lesion masks across all patients (Figure 1), was registered in MNI ICBM152 2009c symmetric space, with the same pipeline as in the subsequent sections on T1w images segmentation (Figure 2). The proportion of lesion masks overlap within each sex group signifies a comparable lesion distribution in females and males despite a disproportion in patient number (17 F, 27 M). Lesions aligned with the typical MCA/ACA territories, extending into the superior temporal gyrus, parietal lobe, central regions, and the inferior and middle frontal gyrus. A limited number of subjects had ACA territory lesions, specifically in the superior frontal gyrus.

### 2.5 Image segmentation and ROIs volume extraction

We designed a dedicated complex segmentation pipeline of T1-weighted images to accurately delineate ipsi and contralesional ROIs at different scales and locations, even in the proximity of lesions. Utilizing the CAT12 segmentation function (CAT12 standalone 1839 version) in expert mode, we first successfully segmented T1-weighted images into tissue probability maps. In order to secure segmentation results and establish a robust pipeline capable of handling lesions, the input T1 image underwent masking with both the brain mask and the lesion mask. Stroke Lesion Correction (SLC) was activated, and the skull-stripping option was deactivated. We employed HD-BET for skull stripping of T1 images (as illustrated in Figure 2) since CAT12 skull-stripping often failed in the presence of lesions. All other parameters were set to their default values. The longitudinal CAT12 pipeline was not employed, as it relies on rigid registration between imaging session, which is unable to yield a suitable average image in the presence of brain growth.

Subsequently, Desikan DK40 and Hammers' atlases (Hammers et al., 2003; Desikan et al., 2006) were registered in each native space. While the CAT12 standard setting effectively ensured a good contralesional hemisphere segmentation and atlas registration, we identified specific mislabelings and errors in the ipsilesional hemisphere due to the lesion-related anatomical distortion.

#### 2.5.1 Global volumes, both hemispheres

First, GM and WM volumes were computed using the white and GM partial volume maps of CAT12. We relied on the Hammers atlas registered in the native subject space to compute three hemispheric volumes: total hemisphere volumes, hemispheric WM, hemispheric cortical GM (subtracting cingulate, cerebellum and sub-cortical structures ROIs initially present in the Hammers atlas from the hemisphere masks). Note that this parcellations did include the lesional volumes.

#### 2.5.2 Lobar volumes, both hemispheres, and cerebellum

Upon conducting individual manual quality checks on the DK40 parcellation of the ipsilesional hemisphere, various mislabelings were identified. These inaccuracies were primarily attributed to the segmentation process making strong anatomical assumptions, introducing labeling biases that even extended to regions distant from the lesion territory. Consequently, we deemed the existing process unsuitable for conducting fine regional analyses in the ipsilesional hemisphere. We thus developed a mono-atlas lobar segmentation method, specifically designed to be resilient in the presence of lesions. To allow analyses on both ipsi- and contralesional hemispheres, a symmetric atlas was necessary.

We utilized the CerebrA atlas, regrouping regions to construct a lobar and cerebellum atlas in native space. This lobar atlas is divided into the frontal, central, parietal, temporal, and occipital lobes. Within this lobar atlas, the pre-, post-, and para-central gyrus were concatenated to form a “central region/lobe,” distinct from the frontal or parietal lobes. The individual deformation fields of each T1 image into MNI ICBM152 2009c symmetric space were generated using ANTs symmetric diffeomorphic registration (Avants et al., 2008), with brain masks and lesion masks serving as registration masks. These deformation fields were subsequently applied to register a Voronoi projection of the CerebrA atlas into individual space. A Voronoi projection assigns to each non-labeled voxel in the volume the same label as the closest labeled voxel.

Following individualized quality checks, this method accurately segmented lobes even in the presence of large lesions (see Supplementary material 1), with the exception of the cingulate cortex, which was excluded due to frequent misalignment of the callosal region. Individual GM probability maps were then employed to compute GM volumes in each lobe and in the cerebellum.

#### 2.5.3 Contralesional cortical GM volumes and cortical thickness

To refine with a state-of-the-art parcellation method the findings obtained in the contralesional hemispheres and to ensure comparability between thickness and volume per region of interest (ROI), we conducted a volumetric analysis of the contralesional hemisphere based on the DK40 atlas. Each GM partial volume map was parcellated according to the closest DK40 label on the individual cortical surface. Subsequently, cortical volume (as presented) and mean thickness (see Supplementary material 2, 3) per DK40 ROI were extracted and subjected to analysis.

#### 2.5.4 Subcortical GM structures

Subcortical GM structures were segmented utilizing Vol2Brain (Manjón et al., 2022). During the upload of T1 images, we provided age and sex as complementary inputs to enhance segmentation performance. While the overall segmentation quality was deemed satisfactory, manual correction was carried out in ~55% of cases, primarily to rectify instances of mis-segmentation in the left hippocampus. Volumes of sub-cortical GM structures were subsequently extracted from both hemispheres.

### 2.6 ROIs exclusion criteria

Given our objective to examine the growth of preserved tissue following an early lesion, it was crucial to select on non-lesional regions to prevent interference in statistical analyses. Consequently, lesioned territories, as described in the Patients section, were excluded by default. Additionally, areas were deemed lesioned if the intersection of the lesion mask with a specific ROI exceeded 2% of the parenchymal volume of that particular ROI. These exclusion criteria were not applied to the global ipsilesional hemispheric volumes (total volume, cortical GM, and WM) to estimate the overall impact of the lesion on the ipsilateral hemisphere.

For further analyses within the ipsilesional hemisphere, the number of subjects thus varied across ROIs due to lesion variability in both location and extent: the insula and parietal lobe were excluded from all analyses as they were lesioned in most patients (>75%). The remaining regions were analyzed in specific patient subsets: frontal lobe (12 F/15 M, and 9 F/11 M at 7 and 16 yo, respectively), central region (6 F/13 M, and 3 F/10 M at 7 and 16 yo, respectively), temporal lobe (11 F/16 M, and 6 F/10 M at 7 and 16 yo, respectively), occipital lobe (15 F/21 M, and 12 F/16 M at 7 and 16 yo, respectively), and subcortical GM structures (10 F/18 M, and 10 F/13 M at 7 and 16 yo, respectively).

In the contralesional hemisphere and the cerebellum (both hemispheres) of patients, no ROI had to be excluded, resulting in the following numbers: 15 F/22 M, and 11 F/17 M at 7 and 16 yo, respectively.

Finally, in the control group (without lesion), the total number of analyzed ROIs was 18 F/16 M and 15 F/16 M at 7 and 16 yo, respectively.

### 2.7 Statistical analysis

#### 2.7.1 Model construction (LMM)

To specifically study the effect of an early lesion and its impact on developmental trajectories, we employed a Linear Mixed Model (LMM) predicting each regional volume. Despite the non-linear evolution of brain region volumes with age, the linearity assumption remains suitable for our study due to our age-constrained dataset, which comprises only two time points. The choice of an LMM enables the incorporation of a “random effect” that specifies subject identifiers, accommodating longitudinal data while also including data from subjects with only one time point.

Modeled effects are the age (volumes and thickness depending on age), sex (males and females do not have the same brain volumes), status (patients vs. controls) and all their interactions to model both typical and lesion-related sex-differences in volumes and developmental trajectories (puberty, etc.). Age was covariant with scanner change, hence the model was not adapted to investigate age effect, but corrected for additive variability due to scanner change (Fortin et al., 2018).

We also modeled a “hemisphere” effect that encodes which hemisphere side (right or left) was in input, because brain regions are known to be asymmetric and/or develop asymmetrically (Williams et al., 2021a,b, 2022). The last modeled effect was the lesion severity score, to correct for intra-group lesion size inhomogeneity with possible slight imbalance between patient subgroups. We chose not to correct for intracranial or equivalent volume as these metrics are covariant with patient status: brain growth is impaired due to missing tissue and gliosis, total brain volume encompasses lesion volume, contralesional hemisphere volume is affected as well, and the lesioned brain exerts different biomechanical forces on the skull growth hence influencing total intracranial volume.

We standardized each ROI metric by subtracting the mean of the population and dividing by its standard deviation. Continuous variables (age, lesion severity score) were also standardized. To avoid covariance with the status effect, the lesion severity score was centered on the patient population, set to 0 for controls, and then standardized for the entire dataset. For categorical variables, the native encoding (dummy coding: [0; 1]) was retained for the status effect, where controls served as the reference group, while the hemisphere and sex effects were coded using “effect coding” ([−1;1] instead of [0,1]).

The LMM formulation for each ROI was as follows:


(1) $$\begin{array}{l} MetricROI\;=\;Age\;*\;Sex\;*\;Status\;+\;Hemisphere\_side\\ \;+\;Lesion\;Severity\;+\;(1\;|\;Participant\_id\_side) \end{array}$$

Where fixed effects are: Age as a discrete variable with two levels: 7 or 16 (before standardization); Sex as a categorical variable with two levels: male (−1) or female (+1); Status as a categorical variable with two levels: patient (+1) or control (0); Hemisphere_side (of the studied volume) as a categorical variable with two levels: left (−1) or right (+1); Lesion Severity as a discrete variable with 5 levels [0:4]. (before standardization, see above); “^*^” Signifies that both main effects and interactions were modeled; “+” Signifies that only main effects were modeled (these effects being considered independent).

1 | Participant_id_side is a random effect, adjusted on intercept value, estimated for each hemisphere of each subject (for example “sub-hcXXX_left”, which correspond to the left hemisphere of control number XXX). Random effects must be independent, homoscedastic and normally distributed. This enabled to have a semi-longitudinal statistical design, while also accounting for individual left-right hemisphere variability.

The model was fitted using the LmerTest (Bates et al., 2015; Kuznetsova et al., 2017) package in R with Residual Maximum Likelihood (REML). Statistical *t*-tests (Satterthwaite's method for degrees of freedom) were performed on the model factors for each effect and interaction, assessing whether they were significantly different from 0. For the model fit output, we chose not to correct for multiple comparisons, but we present each effect value with three different *p*-value thresholds (^*^ <0.05; ^**^ <0.01; ^***^ <0.001).

Betas (effect factors) are expressed as standard deviations of the fitted ROI metric dataset, as each model was fitted on standardized data.

#### 2.7.2 *Post-hoc* tests

To disentangle the multiple interaction effect terms and specifically examine the differences between males and females in patients with reference to their matched controls, *post-hoc* tests were specified using the R module Lmertest (Kuznetsova et al., 2017) and emmeans (Searle et al., 1980). This function compares marginal means (the mean volume estimated by the model for a specific group) between specified subgroups, taking into account associated estimated standard deviations and degrees of freedom (Kenward-Roger method). The resulting *t*-tests assess whether the difference between two subgroups is significantly different from 0. The specified contrasts were designed as follows:

- **[Patients – Controls] for each sex at each age**. This design aimed to minimize the impact of sex and age-related volume variations on compared brain ROI sizes. By comparing each patient population with its corresponding control group, we could estimate the specific effects related to patient status within each sex at each age.
- **[(Patients – Controls)**_**male**_ **– (Patients – Controls)**_**female**_**] at each age**. This contrast tests specifically the interaction effect between sex and status on the marginal means, hence on volume values fitted for age and sex in our cohort. This enabled a targeted investigation of potential sex differences specific to patients with early brain lesions while accounting for the typical sex differences at the group level in our cohort.

In the *post-hoc* tests, the Hemisphere_side and Lesion_severity effects were not explicitly defined in the contrasts. As the LMM model serves as input for the *post-hoc* test function, these effects are regressed and averaged across various subgroups before the comparison of marginal means takes place. Since the models were fit on different volume, using different image processing, they were corrected separately for multiple comparisons. False discovery rate (FDR) correction was applied to correct for three tests on whole-hemisphere metrics, six tests on contralesional hemisphere lobar approach, four tests on ipsilesional hemisphere, seven tests for basal ganglia, and 34 for DK40 atlas results. These numbers correspond to the number of ROIs per approach.

For plotting purposes on the same scale, each marginal means difference was expressed as a relative effect size (proportion of the mean control volume), by dividing the raw volume difference by the mean value of control volume across age and sex for the specific plotted ROI.

## 3 Results

### 3.1 Impact of main clinical effects on regional volumes: multifactorial analysis

**Figure 3:**
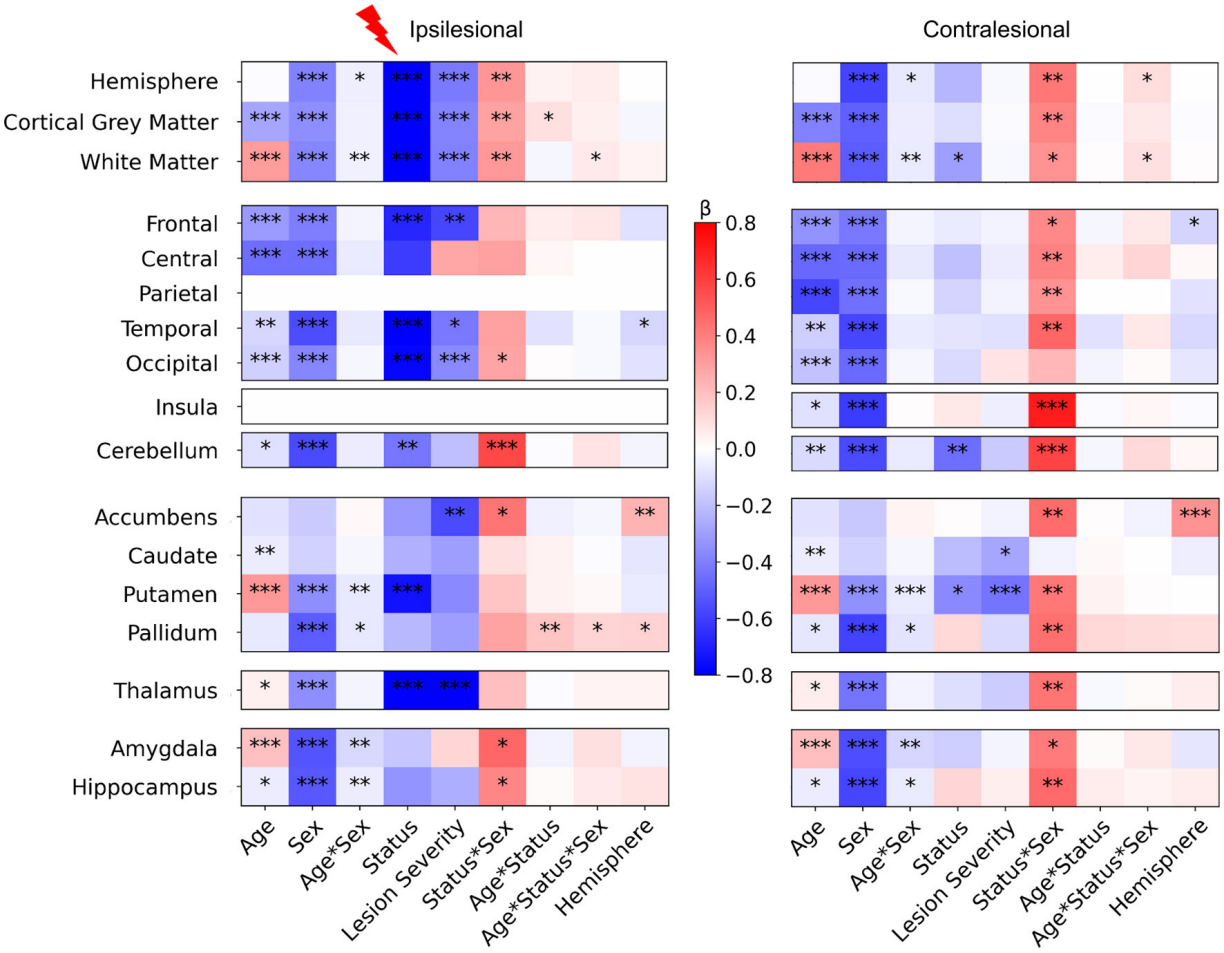
LMM factors values for all regional volumes (fixed effects, main interactions and covariates). *Post-hocs* tests comparing controls to patients mean volume. For plotting purpose, volumes differences are divided by a mean control volume across age and sex (relative effect size) for the shown ROI. Cold colors represent a negative difference (smaller mean regional volume in patients than in controls); warm colors represent a positive difference (bigger patient volumes comparing to controls). *p < 0.05, **p < 0.01, ***p < 0.001 (uncorrected) after False-Discovery Rate correction.

Figure 3 displays the values of each factor and of their interactions in the Linear Mixed Models (LMMs) fitted on each volume of interest. As models were fitted on a normalized dataset, each factor was expressed as a proportion of its standard deviation. Associated statistical tests assessed whether the factor value significantly differed from 0. *P*-values in Figure 3 are uncorrected. Parietal lobe and insula in the ipsilesional hemisphere were excluded as they were lesioned in most patients. For further description of raw total and hemisphere volume values in each age and sex group, please refer to Supplementary material 4.

#### 3.1.1 Main effect of age

The model fit results showed consistent changes between 7 and 16 years of age for cortical GM (negative) and WM trajectories (positive), as commonly described in the literature (Bethlehem et al., 2022; Rutherford et al., 2022), and in most basal ganglia volumes (Wierenga et al., 2014). However, our study design aimed at comparing groups within age category rather than between ages.

#### 3.1.2 Main effect of sex

The results also showed consistent data with typical sex differences, recently confirmed by normative analysis (Wierenga et al., 2014; Bethlehem et al., 2022; Rutherford et al., 2022). Sex factors were significantly negative in nearly all ROIs, confirming smaller mean volumes in females than in males across all groups, a well-documented difference in the literature.

#### 3.1.3 Interaction between age and sex

The Age^*^Sex interaction was weak but significant, in both ipsi and contralesional hemispheres, in the hemispheric WM volume (with a marginal effect on the whole hemisphere volume), and in some subcortical structures (putamen, pallidum, amygdala, and hippocampus). This may relate to the subtle asynchrony in GM volume developmental trajectories between males and females (Giedd et al., 2015; Bethlehem et al., 2022). No such effect was seen in the cerebellum.

#### 3.1.4 Asymmetry (hemisphere side) covariate

Model fits showed very limited effects of hemispheric side in ipsi and contralesional hemispheres, with a small negative effect on some lobar regions (temporal ipsilesional, and frontal contralesional), indicating slightly bigger left than right volumes, and positive tendencies in pallidum and accumbens, indicating bigger right than left volumes. These regional differences were however subtle.

#### 3.1.5 Differences between patients and controls (status) and effect of lesion severity

Interestingly, the effect of status (patient vs. control) and of lesion severity were different between hemispheres. In the ipsilesional hemisphere, as expected, the whole hemisphere, WM, and cortical GM volumes (that include the lesion mask) showed a strong negative effect size, i.e.. smaller volumes in patients than in controls, with the effect of lesion severity adding to smaller volumes of intact brain. Indeed, in cortical regions distant from the lesion too (frontal, temporal and occipital lobes), patients had smaller volumes than controls, regardless of age, sex, or hemisphere, with an effect of lesion severity, suggesting an ipsilateral growth restriction remotely from the lesion and depending on severity. In subcortical regions, the putamen and thalamus volumes showed a similar behavior confirming previous data showing altered cortico-subcortical connectivity, that was correlated with lesion severity in the thalamus. By contrast, in the contralesional hemisphere, there were limited volume differences between patients and controls, with only hemispheric WM and putamen volumes being significantly smaller in patients. Only the contralesional reduction of the putamen volume was related to lesion severity. Pallidum exhibited a similar tendency, although the status effect was not significant, which might indicate that only patients with the biggest lesions showed volume loss.

Eventually, cerebellar GM volumes of both hemispheres were significantly lower in patients, with no obvious impact of lesion severity.

There was a very weak interaction between status and age in the ipsilesional hemispheric GM volume, but not in ispilesional regions distant from the lesion, except the pallidum. This may indicate minimal volume trajectory difference between 7 and 16 years between patients and controls (see *post-hoc* tests below).

#### 3.1.6 Interaction between status and sex

This interaction questions whether the volume differences between patients and controls depend on the subject's sex, regardless of known typical differences due to age and sex (main effects). Interestingly we found significant interactions in both hemispheres, however much more pronounced in the contralesional hemisphere.

In the ipsilesional hemisphere, the Status ^*^ Sex interaction was significantly positive for hemisphere-wise volumes, and in the occipital lobe only at the lobar scale. Among subcortical GM volumes, it was also significantly positive in accumbens, amygdala, and hippocampus volumes. In the contralesional hemisphere however, the Status ^*^ Sex interaction effect was significantly positive in all volumes except the occipital lobe and caudate nucleus.

The Status ^*^ Sex interaction was significantly positive in both cerebellar hemispheres. Adding age in the interaction (Age ^*^ Sex ^*^ Status) showed a very weak positive effect in ipsilesional WM and pallidum, and in contralesional whole hemisphere and WM volumes.

### 3.2 Comparison between patients and controls according to sex (*post-hoc* tests)

**Figure 4:**
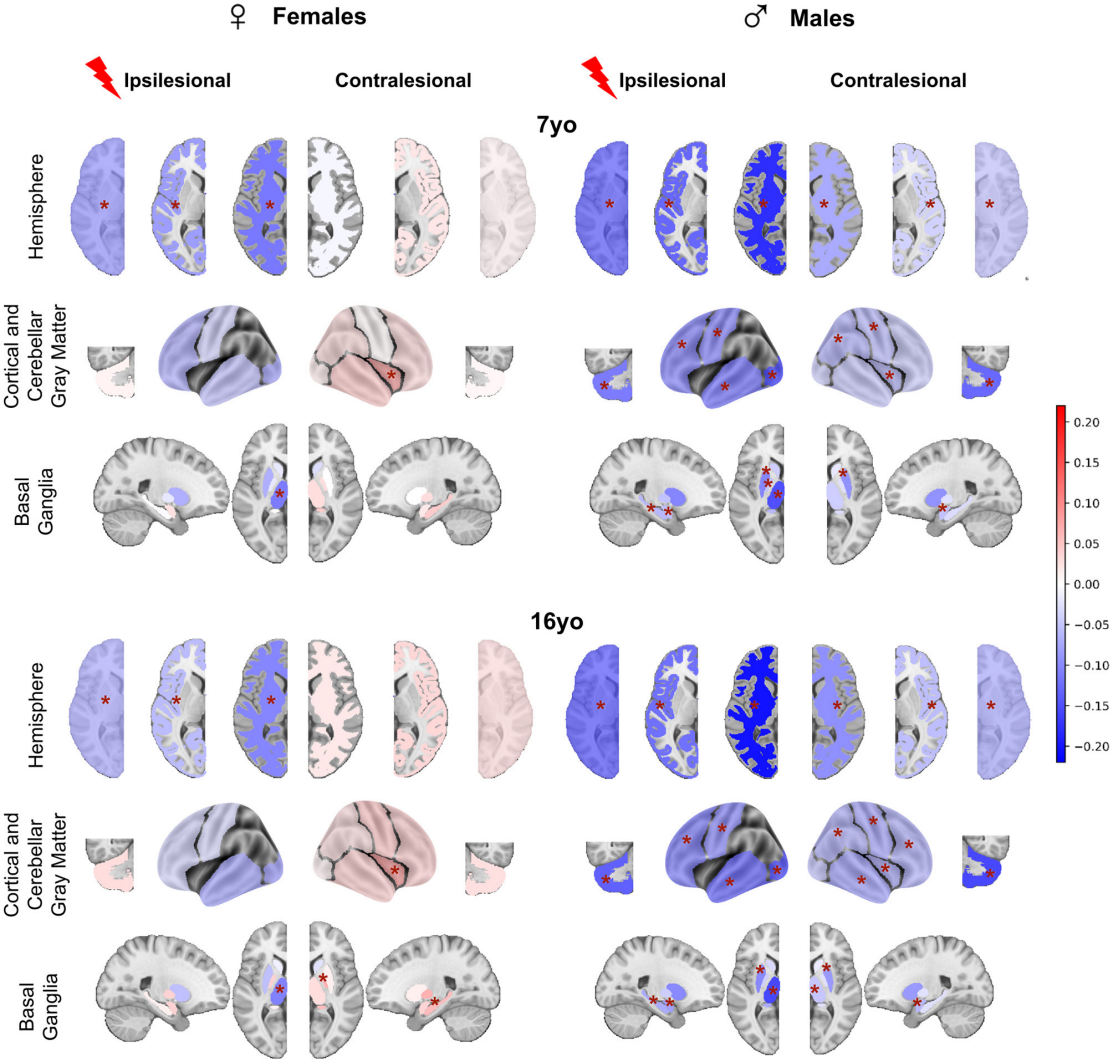
*Post-hoc t*-tests comparing patients and controls for each age and sex (patients—controls). For plotting purpose, volumes differences are divided by a mean control volume across age and sex (relative effect size) for the shown ROI. Cold colors represent a negative difference (smaller mean regional volume in patients than in controls); warm colors represent a positive difference (bigger patient volumes comparing to controls). *p < 0.05 after False-Discovery Rate correction.

To further explore the Status^*^Sex interaction described above, we compared mean brain regional volume differences between patients and controls in all sex and age groups (Figure 4). All raw differences in volumes were divided by a mean control volume across ages and sexes of the corresponding ROI to allow better figure readability (color and effect size normalization). Therefore, all differences are expressed as proportions of a reference control volume. A negative difference (blue color) means that the mean regional volume was smaller in patients than in controls. If positive (pink/red color), patient volumes were bigger than control ones.

#### 3.2.1 In females

At 7 yo, we found very limited differences between female patients and controls. While ipsilesional hemispheric WM and cortical GM volumes were significantly lower in patients than in controls (−5.7 to 11.7% of control mean volume), there were no significant volume differences of intact lobes and of cerebellar GM volume between female patients and controls. This suggests that the reduction of hemispheric volumes reflected the lesion extent only. Among ipsilesional subcortical GM structures, only the thalamus volume was smaller than in the controls (−11.2% of the mean control thalamus volume). This thalamus volume reduction reflects the well-known dysconnectivity of the cortico-subcortico-thalamic loop (Xia et al., 2021). Interestingly, female patients contralesional hemisphere volumes were not significantly different from controls. On the regional analysis, patient contralesional insula volumes were even significantly larger than in controls (+5.6%). No difference was found elsewhere, including in the cerebellum. At 16 yo, the pattern was similar to that of 7 yo. The only minimal change consisted of bigger contralesional pallidum and hippocampus in female patients than in controls (+6.5 and 5.8%). These observations suggest a notable resilience of brain growth after an early brain lesion in females, and even maybe some compensation mechanism, whose cellular mechanisms cannot be informed by mere volumetric descriptions (synaptic densities, myelination ? …).

#### 3.2.2 In males

By contrast, we found significant volume reductions in male patients as compared to male controls, in both hemispheres, and at both ages. At 7 yo, ipsilesional hemisphere, WM, and cortical GM volumes were significantly smaller than controls (−12.1 to −20.3%). All ipsilesional lobes and subcortical GM structures distant from the lesion showed significant volume reductions in patients compared to controls (lobes = −9.3 to −19.6%, hippocampus = −6.1%, amygdala = −5.3%, putamen= −10%, pallidum= −5.1%, and thalamus = −14%). This demonstrates a volume reduction of all ipsilesional remote structures in addition to the direct lesion effect.

In the contralesional hemisphere, whole hemisphere, WM, and cortical GM volumes were also significantly lower in male patients than in controls (−3.9 to −9.6%). Lobar analysis showed significantly lower volumes in contralesional parietal lobe, central region and insula (−5.6%), as well as in amygdala (−5%) and putamen (−9.2%). Cerebellum was smaller on both sides (−10%). At 16 yo, the pattern was essentially identical to that of 7 years in the ipsilesional hemisphere. In the contralesional hemisphere, minimal changes were seen as volume reduction also reached significance in the frontal and temporal lobes (−5.8 and 7.4%), in the thalamus (−4.9%), and the hippocampus (−2.6%).

### 3.3 Comparison between male and female patients accounting for typical sex differences

**Figure 5:**
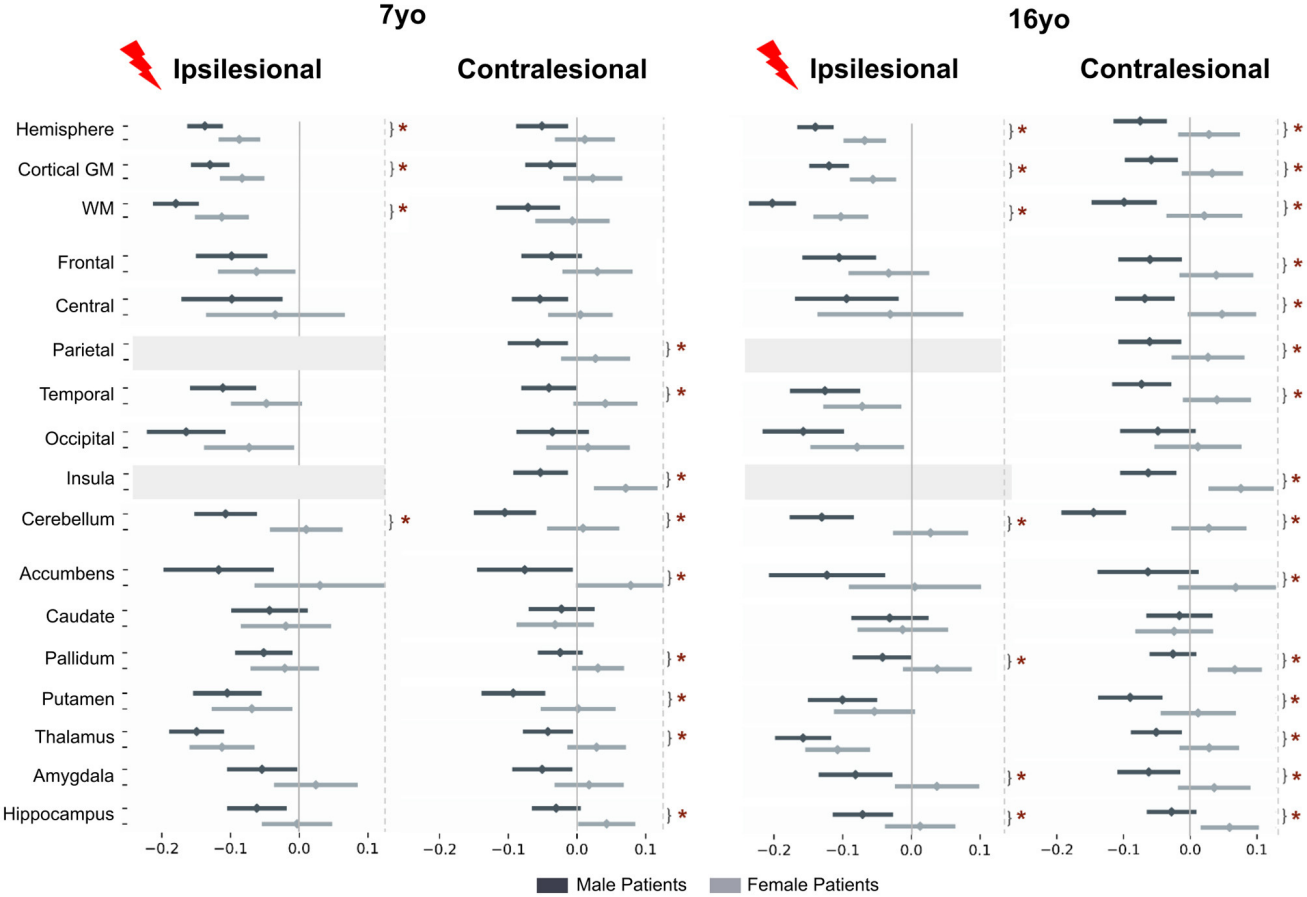
Patient to control volume differences (mean and std) for each sex and each age. *Post-ho*c *t*-tests assess if interaction product [(patient-control) male – (patient-control) female] is different from 0. Tests are performed on marginal means, hence typical male to female differences in the control group are residualized in the contrast. For plotting purpose, volume differences are divided by a mean control volume across age and sex (relative effect size) for the shown ROI. Upper dark gray lines represent male results, light gray the female ones. *p < 0.05 after False-Discovery Rate correction.

To assess whether these sex differences according to the clinical status were really significant, while accounting for the known differences in typical subjects, we used the interaction product contrast (*[(patient – control) male – (patient - control) female]*) (Figure 5). In nearly all ROIs, volumes were smaller in males than in females, reaching significance in almost all contralesional regions, and also in a number of ipsilesional ones, with an identical pattern at 7 and 16 years of age. Ipsilesional hemispheric volumes were significantly smaller in male than in female patients at both ages (on the average −2.5 and −3.5% at 7 and 16 yo, respectively). This difference is independant from lesion severity, as the model already corrected for it. In ispilesional lobes distant from the lesion, male and female cortical GM volumes were not significantly different, although the trend in all regions was toward smaller volumes in males. Ipsilesional pallidum, hippocampus and amygdala were also significantly smaller in male than female patients at 16 yo (−3.9, −4.1, −5.9%, respectively).

In the contralesional hemisphere, the male-female differences were even more pronounced, especially at 16 yo. Hemispheric volumes were significantly smaller in males at 16 yo only (−5.8%). The lobe-wise pattern was the same at both ages: parietal (**–**4.1%), temporal (−4.1 to **–**5.4%) and insula (**–**6.2 to **–**6.7%), or at 16 yo only (frontal lobe and central region, **–**5.6 and **–**4.8%, respectively). Contralesional accumbens (**–**7.7, **–**6.3%), pallidum (**–**2.7, **–**4.5%), putamen (**–**4.7, **–**4.9%), thalamus (**–**3.5, **–**3.8%) and hippocampus (**–**3.6, **–**4.2%) were all significantly smaller in male than in female patients at both ages, and amygdala volume difference was significant only at 16 yo, **–**4.7%). Cerebellum was significantly smaller in male than in female patients at both ages and on both sides (ispilesional: **–**5.8 and **–**7.9%, and contralesional: **–**5.8 and **–**8.3% at 7 and 16 years of age, respectively).

### 3.4 Regional study of the contralesional hemisphere in the Desikan-Killiany (DK40) atlas

**Figure 6:**
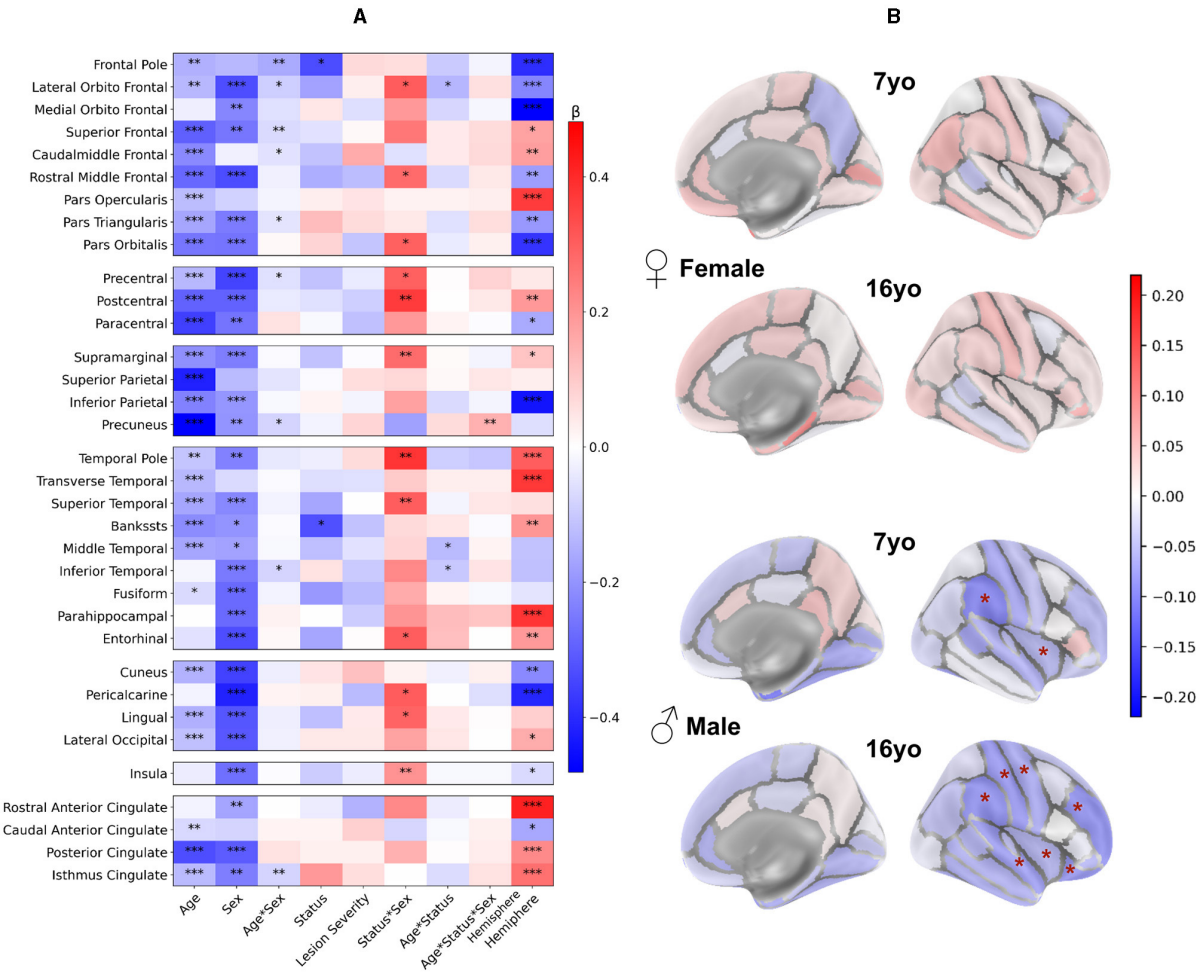
Contralesional volumes of patients compared to controls in 34 ROIs of Desikan-Killiany parcellation: LMM model and *post-hoc* tests. Patient volumes are compared to the mean of both hemispheres corresponding values in controls. **(A)** LMM coefficients values for each region of the DK40 (fixed effects, main interactions, and covariates). *p < 0.05, **p < 0.01, ***p < 0.001. Plotting parameters are the same as in Figure 3. **(B)** *Post-hoc* tests comparing patients and controls for each age and sex (patient – controls). Displayed *p*-values are FDR-corrected (* <0.05). Parameters are the same as Figure 4.

Beyond this relatively coarse analysis of ipsi and contralesional hemispheric and lobar volumes, we aimed at a finer contralesional ROIs parcellation in order to test whether regions homotopic to the lesion would show a particular vulnerability in terms of cortical GM growth. This hypothesis stems from the well-known diaschisis that affects regions connected to a lesioned territory, especially through callosal interhemispheric homotopic fibers (Pretzel et al., 2022). To investigate this, we designed a finer contralesional parcellation, in DK40 atlas, and applied the same model and analysis grid (Figure 6).

#### 3.4.1 Impact of the clinical variables

Figure 6A displays the coefficients obtained from the models. Consistently with the lobar results, the Age and Sex effects were negative in most regions, indicating lower volumes at 16 than at 7 yo and lower volumes in females than males. Age^*^Sex effect was rarely significance (uncorrected) with a small effect size. Status and lesion severity had little to no effect. We confirmed the positive Status^*^Sex interaction effect in several regions, suggesting a divergence from the norm in male vs. female patients, like in the previous analysis.

Age^*^Status interaction effect showed a similar behavior than in previous analysis, but reached significant (uncorrected) in a few regions (lateral orbitofrontal gyrus, middle and inferior temporal gyrus). This may indicate a slightly more pronounced volume decrease between 7 and 16 yo in patients than in controls. As for previous results, no Age^*^Sex^*^Status interaction was significant.

However, unlike lobar scale results, the Hemisphere effect was significant in many ROIs. Most effects were in line with recent studies on brain asymmetries studied with similar brain parcellation (Williams et al., 2022).

#### 3.4.2 Comparison between patients and controls per sex group

In Figure 6B, sex and age-paired comparisons between patients and controls revealed distinct patterns: In females, there was no difference in any regional volume between patients and controls at both ages. In males, the supramarginal gyrus and insula were significantly smaller in patients than in controls (**–**11.6 and **–**5.9%, respectively) at 7 yo. At 16 yo, volumes were smaller in superior temporal gyrus (**–**9.1%), supramarginal gyrus (**–**10%), postcentral (**–**9.5%) and precentral gyrus (**–**7.6%), rostral middle frontal gyrus (**–**10%), lateral orbitofrontal (**–**9.5%), and insula (**–**6%). Thus, this finer parcellation confirmed the trends observed previously, with very little deviation from the typical trajectories in females, and decreased volumes in male patients, mostly in perisylvian regions, mirroring the lesion distribution in the opposite hemisphere.

When looking at male to female volume differences specific to the patient status effect (see Supplementary material 5) we found similar tendencies in DK40 atlas as with the lobar atlas in almost all regions, consistently at 7 and 16 yo. However, no differences survived the FDR correction. This was probably due to several reasons: (i) As ROIs were smaller, the data variability was bigger compared to the observed effect size; (ii) Volume differences between patients and controls were smaller than on the lobe wise analysis, with lower effect size, and as such, less significant results; (iii) The multiple tests correction was more stringent given the number of tests with regard to the limited number of patients.

We confirmed these sex differences by exploring two alternative models: a sex by sex analysis, and a residualised approach, where we fitted a Age ^*^ Sex Generalized Model before fitting an adapted Linear Mixed Model **(**see Supplementary material 6–11).

The same analysis was performed on mean cortical thickness (see Supplementary material 1, 2) in DK40. Model factors exhibited more dispersed tendencies, leading to inconclusive results in *post-hoc* tests. This inconclusiveness primarily stemmed from smaller effect sizes, typically below 3%.

## 4 Discussion

This study is the first step of a larger protocol aiming at studying the long-term plasticity of the brain during childhood and adolescence after an early focal brain injury, through a combination of multimodal clinical and MRI markers. In this paper, we present the first longitudinal multifactorial MRI morphometry analysis of the long-term sequels after a neonatal stroke, accounting for developmental and clinical factors (age, sex, lesion side and extent). Relying on a carefully curated cohort of subjects with unilateral NAIS studied longitudinally within specific age ranges (7–8 and 16–18 years of age), we underlined the impact of the different factors on the ipsi- and contra-lesional cortical, subcortical and cerebellar GM distant from the lesion growth trajectories, with reference to age-matched controls.

We designed a robust image processing and segmentation pipelines capable of handling the lesion-related anatomical modifications. Volumes were extracted at different scales (from hemispheric to regional parcels). Age-matched controls were recruited for normative purposes, with more than half of those also studied longitudinally. Utilizing a Linear Mixed Model with numerous regressors, we obtained reproducible and congruent results across different morphometry pipelines and granularity levels.

We found an overall reduction of most volumes distant from the lesion in patients compared to controls, with different patterns in ipsi- and contra-lesional hemispheres depending on sex (after controlling for all other variables, including lesion severity). As expected, volumes distant from the lesion were more reduced in the ipsi-lesional hemisphere than in contra-lesional one, and depended on lesion extent, reflecting intrahemispheric disconnectivity. The ipsilesional volume reductions were slightly more pronounced in males than in females. In the contralesional hemisphere, sex differences were much more pronounced, with males exhibiting smaller regional cortical (and some subcortical) GM volumes, while females showed little deviations from the typical trajectories. When utilizing finer parcellations, the volume reductions were mostly seen in homotopic regions to the lesion within the contralesional hemisphere, suggesting specific interhemispheric homotopic disconnectivity after a unilateral NAIS. The hippocampus/amygdala volumes followed a similar pattern with reduction in males, and stability/increase in females. Bilateral cerebellar gray matter was reduced in males only. Interestingly, the impact of puberty was weak, as volume differences between patients and controls exhibited similar magnitudes at age 7 and 16, suggesting that most, if not all, deviations had occurred in early childhood (before 7 yo).

Among previous morphometric studies on perinatal stroke, very few investigated specifically sex differences in volume trajectories. Most of them controlled for sex ratio differences in each group composition before conducting analysis without including sex as covariate (Gold and Trauner, 2014; Dinomais et al., 2015; Craig et al., 2019a,b; Hassett et al., 2022; Ilves et al., 2022; Shinde et al., 2023; Vaher et al., 2023). One study explicitly stated controlling analysis for sex before not including it as covariable (Al Harrach et al., 2019), and two compared each patient to an age and sex paired control group (Moses, 2000; Stiles et al., 2009). To our knowledge, our study is the first one tackling specifically sex differences in brain volumetry after a neonatal stroke.

### 4.1 GM volumes reduction in ipsilesional hemisphere

In the ipsilesional hemisphere, GM volumes distant from the lesion were consistently smaller in patients compared to controls, in both sexes and ages, independently of the stroke side and severity. Additionally, these volumes exhibited a dependence on lesion extent in cortical lobar regions (except the central region) and in the thalamus. *Post-hoc* tests revealed significant differences in lobar parcels for males. The total hemisphere volumes (WM and cortical GM volumes) do include the lesioned area (that is accounted for in the lesion severity regressor). Thus, differences between patients and controls at the hemispheric level can be explained by the difference of volumes distant from the lesion as seen in each lobe, adding to the lesion effect. Moreover, subcortical GM volumes were also altered, with thalamic volumes significantly smaller in both sexes, in line with (Craig et al., 2019a; Vaher et al., 2023).

This pattern likely relates to distributed intrahemispheric disconnectivity, leading to changes in brain areas functionally connected but structurally remote from the primary lesion (i.e., diaschisis), and resulting in the reduction in GM volumes distant from the lesion in all associative territories with long range connectivity. The exception of the central region might come from the peculiar connectivity of this primary projection cortex that essentially connects with subcortical and cerebellar regions. In premature babies, WM lesions such as periventricular leucomalacia (PVL) have been shown to lead to reduced GM volumes at term (Inder et al., 1999). Previous diffusion studies of NAIS have also consistently identified diaschisis in GM and WM, such as in the thalamus or the corpus callosum (Srivastava et al., 2019), and beyond corpus callosum (Pretzel et al., 2022). As expected, we also found a global reduction in hemispheric WM (beyond the lesion volume), but more detailed study of WM bundles will help to correlate the altered structural connectivity pattern following NAIS with GM volumes. Thalamus volume was significantly smaller in both sexes, in line with (Craig et al., 2019a; Vaher et al., 2023), its central position making it susceptible to diaschisis, as described by previous analyses (Carrera and Bogousslavsky, 2006) linking regional thalamic injuries to many cortical lesion syndromes.

While diaschisis has been extensively studied in adult stroke using older imaging modalities, the contemporary definition of “connectomal” diaschisis (Carrera and Tononi, 2014), made possible by advanced imaging modalities, presents a promising avenue for understanding the clinical impairment and recovery after NAIS (Shi et al., 2019; Umarova and Thomalla, 2020; Siddiqi et al., 2022).

### 4.2 Differentiated pattern of GM volumes in the contralesional hemisphere

In the contralesional hemisphere, only male patients exhibited significantly smaller GM volumes than controls. This pattern was reproduced by the DK40 parcellation, albeit more focused on homotopic regions mirroring the lesion. By contrast, female patients did not deviate from the controls values and had an even slightly larger insula than controls, that could illustrate sex differences in compensatory mechanisms.

Sub-cortical analysis revealed smaller putamen and amygdala at both ages, along with a smaller thalamus at 16 years in male patients. Female patients, on the other hand, exhibited larger hippocampus and pallidum than controls at 16 yo. Patient sex-related differences were significant only at 16 years, in hippocampus, amygdala and putamen. Lower volumes were found in pallidum and hippocampus in perinatal AIS (Ilves et al., 2022), and hippocampus, amygdala and putamen lower volumes correlated with poorer hand function. To our knowledge, the sex-difference we found in subcortical GM has never been described before.

These congruent and robust results at different scales and with different atlases indicate long-term contralesional differences in GM growth between male and female patients after NAIS. Specific *post-hoc* tests yielded significant results at both ages, encompassing whole hemisphere volumes, all lobes but the occipital, and almost all subcortical volumes but the caudate. However, significance was not reached when using the DK40 parcellation after correcting for multiple comparisons.

Divergences between these two scales can be attributed to various methodological factors. Inter-individual variability is more prominent in smaller regions, where subtle segmentation errors also contribute to a larger variability. Smaller regions also tend to exhibit greater asymmetry, particularly during development. Regional asymmetries are a key feature of mature brain, and start during prenatal development (Chi et al., 1977; Toga and Thompson, 2003; Maingault et al., 2016; Williams et al., 2022). Brain maturation unfolds asynchronously, with finer associative functions maturing later than primary ones, and with a precise spatiotemporal sequence. In our model, this asynchrony was accounted for by the hemisphere regressor, but this correction may be improved, as recent large scale studies pointed sex differences in these asymmetries (Williams et al., 2022). Finally, significantly more tests were conducted on the DK40, making multiple tests correction more stringent, given our limited sample size (although the largest sample to date of NAIS patients studied longitudinally over 17 years).

### 4.3 Cerebellum morphometry

Consistently with prior results, we observed smaller cerebellar hemispheric GM volumes in male patients compared to controls (but not in females) at both ages and in both hemispheres. In this study, we did not intend to study specifically cerebellar asymmetries nor subcerebellar parcels, and no differences were identified between ipsi- and contra-lesional hemispheric cerebellar GM volumes in any group. Cross cerebellar diaschisis has been observed repeatedly (Dinomais et al., 2012; Craig et al., 2019b). The role of the ipsilesional cerebellar hemisphere volume alterations on cognitive outcome after perinatal stroke has been recently suggested (Craig et al., 2019b).

### 4.4 Sex related differences

Overall, our study shows a significative difference between males and females in the brain developmental anatomical evolution, especially within the contralesional hemisphere, following the dysconnectivity induced by an focal neonatal brain lesion. Numerous factors may contribute to these sex differences on brain architecture and organization (DeCasien et al., 2022), including chromosomal differences and sex bias in autosomal gene expression (Shi et al., 2016). Our results are in line with the vast literature validating the neurobiological differences between males and females with respect to the response to brain injuries and in particular the early vulnerability of the male brain (Smith et al., 2014; Charriaut-Marlangue et al., 2018; Kelly et al., 2023). While the mechanisms underlining this early sex difference remain unclear, several biological processes have been suggested: vulnerability to oxidative stress, modulation of cell death, regulation of microglial activation and gonadal hormones exposure across developmental stages (Charriaut-Marlangue et al., 2018).

As our results indicate a same order effect size at 7 and 16 years, we infer that most observed differences occurred before 7 years of age and were minimally impacted by the physiological development and by the shift in sex steroid exposure during puberty. This suggests that sex might influence early post-injury plasticity and other compensation mechanisms. Several non-exclusive hypotheses can be proposed. One is hormonal, given the differences in prenatal and early post-natal exposure to gonadal hormones, due to a sex-difference on the hypothalamus-pituitary gonadal axis activation. This induces in both sexes a transient “minipuberty” (Becker and Hesse, 2020), that could impact several neurobiological mechanisms including the neuroinflammatory response to the lesion. Another hypothesis involves differences in brain structure maturation at birth between males and females. Recent studies have shown that microglia cells are sexually dimorphic and play a role in sexual differentiation, but they also intervene in the immune response to brain lesions (VanRyzin et al., 2020).

Sex related differences have been described in preclinical inflammation models showing an upregulated immune response and increased microglial activation in males following neonatal hypoxia (Kelly et al., 2023). Also, the materno-fetal innate immune signaling shows a more prominent inflammation in males than in females, with sex specific decreased myelination, decreased WM thickness, microgial depletion in the corpus callosum, and neuronal apoptosis (Giraud et al., 2017; Allard et al., 2019). In a placental study in twins, male placentas were shown to have more inflammation than girls (Jahanfar and Lim, 2018). Sex-specific placental and fetal pro-inflammatory responses are in keeping with the higher susceptibility of males for preterm birth, early brain injuries, and neurodevelopmental disorders such as CP, and ASD. Eventually, sex differentiated segregation of genetic polymorphisms might contribute to this pattern (Dewing et al., 2003; McCarthy et al., 2017; Raznahan et al., 2018). Independently of the underlying mechanisms, our observations concur to an early deviation from typical trajectory in males, while females seem to be more resilient, and to present less morphological changes in later childhood and adolescence.

### 4.5 Functional correlates

The relationship between atypical brain size/growth and the variability of neurocognitive outcomes remains unclear. In most cases, cognitive deficits suggest cortical and/or subcortical neuronal dysfunction, that often translates in reduced volumes/growth, especially in case of characterized perinatal brain lesions (i.e., hypoxic-ischemic encephalopathies). On the other hand, reduced volumes do not always translate into clinically relevant correlates, as shown by the large inter-subject variability of brain volume in the typical population (Bethlehem et al., 2022; DeCasien et al., 2022; Rutherford et al., 2022).

Beyond the sex ratio in the occurrence of NAIS (two males for one female) (Chabrier et al., 2016), data on sex related functional/clinical consequences correlated to the morphological brain changes after early brain lesions remain scarce. For example, as some volumes are larger in female patients than controls (e.g., insula, hippocampus), it may be tempting to conclude that females compensate anatomically for their lesions, whereas males do not. However, this hypothesis might be somewhat simplistic, as previous work has highlighted volume variations outside the norm that do not correlate with clinical variables (Craig et al., 2019b). In general, the anatomo-functional correlations between morphometry and clinical scores remain deceiving in a number of situations (Gur and Gur, 2017; Peper et al., 2020). In previous literature, no clear sex differences have emerged so far on behavioral analysis, suggesting that the contribution of sex remains moderate in comparison to major clinical variables such as lesion extent and co-morbidities. More thoughtful designs may address this issue in depth.

### 4.6 Methodological considerations

This study is a pioneering endeavor, distinguished by its reliance on a partly longitudinal cohort of both patients and controls. This unique design facilitates the utilization of a Linear Mixed Model, incorporating a linear model of age, given our two strictly defined age ranges. This enhances the statistical power of the analyses, enabling a multifactorial investigation that accounts for various developmental factors, for the first time in this pathology. Notably, to our knowledge, this is the first comprehensive exploration of the ipsilesional hemisphere and of sex effects in this context.

Our segmentation methodology proved robust to the presence of lesions, and we expect that improved volumetric parcellation will contribute to more reliable results in subsequent diffusion and functional imaging analyses. Rigorous quality checks were applied to the images to ensure the absence of artifacts that could corrupt the analyses. Lesions were characterized based on clinical and imaging criteria, ensuring a highly reproducible phenotype and restricting its clinical and structural variability. To further address lesion variability in the model, we designed a specific lesion severity score. We developed a methodology to strictly select regions distant from the lesion and secure analyses on the cortical regions of the ipsilesional hemisphere. We accounted for brain local asymmetry directly in the model as well as in the imaging pipeline by using symmetric templates. The use of complementary image processing and segmentation methodologies resulted in consistent tendencies increasing confidence in the findings.

Some limitations should however be acknowledged in the current study. While we did our best to avoid selection biases and checked the different subgroups clinical characteristics, we cannot fully assert a perfect generalizability of our results to the NAIS population.

In addition to the fact that NAIS is a rare disease, the AVCnn cohort is a unique cohort that, from a clinical perspective, present a large number of subjects studied longitudinally over nearly 20 years. From an imaging and statistical perspective however, this sample remains relatively small, especially for the female subgroup. Although the statistical models used estimate standard errors and degrees of freedom based on subgroup sizes, the limited population of 16 yo females with a right lesion may not be generalizable. We however remain confident in our results on the female subgroup, given the excellent coherence of the results at age 16 with those at age 7 with more subjects.

Also, because of the limited sample size, we could not include more variables in the LMM analysis. To alleviate this possible confound, we checked beforehand that there were no differences between females and males characteristics, in particular for variables that may induce a bias, including the presence of epilepsy (Vaher et al., 2023), cerebral palsy (Dinomais et al., 2015), and the family socio-economic status

In this long-term longitudinal study, we could not avoid a scanner bias due to MRI scanner change between 7 and 16 years of age (covariance with age). This problem was solved by regressing the scanner bias in conjunction with the age effect in the model.

Despite our best efforts to design our registration pipeline, and a thorough quality check we cannot exclude some residual misregistration in the ipsilesional hemisphere, due to non-linear brain growth around the lesion scar. However, this effect is unlikely to affect the results in remote regions and it has been mitigated in the close vicinity of the lesion by our stringent exclusion criteria of lesioned ROIs. Lastly, given the complexity of hemispheric asymmetries specially in the presence of a lesion, lesion side effect might be imperfectly accounted for by the hemisphere side regressor.

## 5 Conclusion

This first longitudinal study on long-term global and regional brain growth after NAIS highlights sex differences in contra-lesional brain volumes. Notably, while females did not deviate from controls, males exhibited smaller volumes of gray matter in both cortical and subcortical compartments, and in the cerebellum. These sex related differences were apparent in early childhood, and puberty did not change the pattern.

Thus, beyond the known increased frequency of early setbacks in males, this study demonstrates intrinsic sex-related differences in brain growth *after* the initial event, at the expense of males that show an early restricted growth compared to male controls. The biological mechanisms of male brain response to injury results in a multifactorial cascade that takes place in early childhood, with a continuing deviation from the typical pattern that is not much influenced by puberty. Further work on detailed assessment of white matter growth and connectivity will provide a more comprehensive understanding of the neurobiological underpinnings of sex-related differences in the aftermath of NAIS.

How this translates into clinical sex-related differences will also need to be further detailed.

Eventually, this study underscores the critical importance of accounting for typical sex-related volume differences in the developing brain where the temporality and amplitude of brain maturation follow a non-linear trajectory, varying by sex and brain region.

## Data availability statement

The raw data supporting the conclusions of this article will be made available by the authors, without undue reservation.

## Ethics statement

The studies involving humans were approved by Data at 7 years of age: PHRC régional n80308052, and PHRC interrégional n81008026—eudract number 2010-A00329-30 and Data at 16 years of age: ethical approval ID-RCB 2020-100106-33 (CPP Devine). The studies were conducted in accordance with the local legislation and institutional requirements. Written informed consent for participation in this study was provided by the participants' legal guardians/next of kin.

## Author contributions

P-YP: Conceptualization, Data curation, Formal analysis, Investigation, Methodology, Software, Validation, Visualization, Writing – original draft, Writing – review & editing. YL: Conceptualization, Data curation, Formal analysis, Methodology, Software, Supervision, Visualization, Writing – review & editing. SB: Data curation, Investigation, Methodology, Software, Writing – review & editing. LD: Conceptualization, Data curation, Investigation, Methodology, Writing – review & editing. EP: Conceptualization, Data curation, Investigation, Methodology, Writing – review & editing. IB: Conceptualization, Investigation, Methodology, Validation, Writing – review & editing. DB: Methodology, Software, Writing – review & editing. SN: Data curation, Validation, Writing – review & editing. ED: Methodology, Software, Writing – review & editing. MD: Conceptualization, Data curation, Funding acquisition, Investigation, Methodology, Writing – review & editing. MC: Writing – review & editing, Conceptualization, Methodology, Supervision, Visualization. LH-P: Formal analysis, Funding acquisition, Investigation, Methodology, Project administration, Supervision, Validation, Visualization, Writing – review & editing, Conceptualization, Data curation.
