# Supplementary figures and images for: Brain growth until adolescence after a neonatal focal injury: sex related differences beyond lesion effect
Source: Front Neurosci. 2024 Aug 23;18:1405381. doi: 10.3389/fnins.2024.1405381 (PMC11378422; doi:10.3389/fnins.2024.1405381)

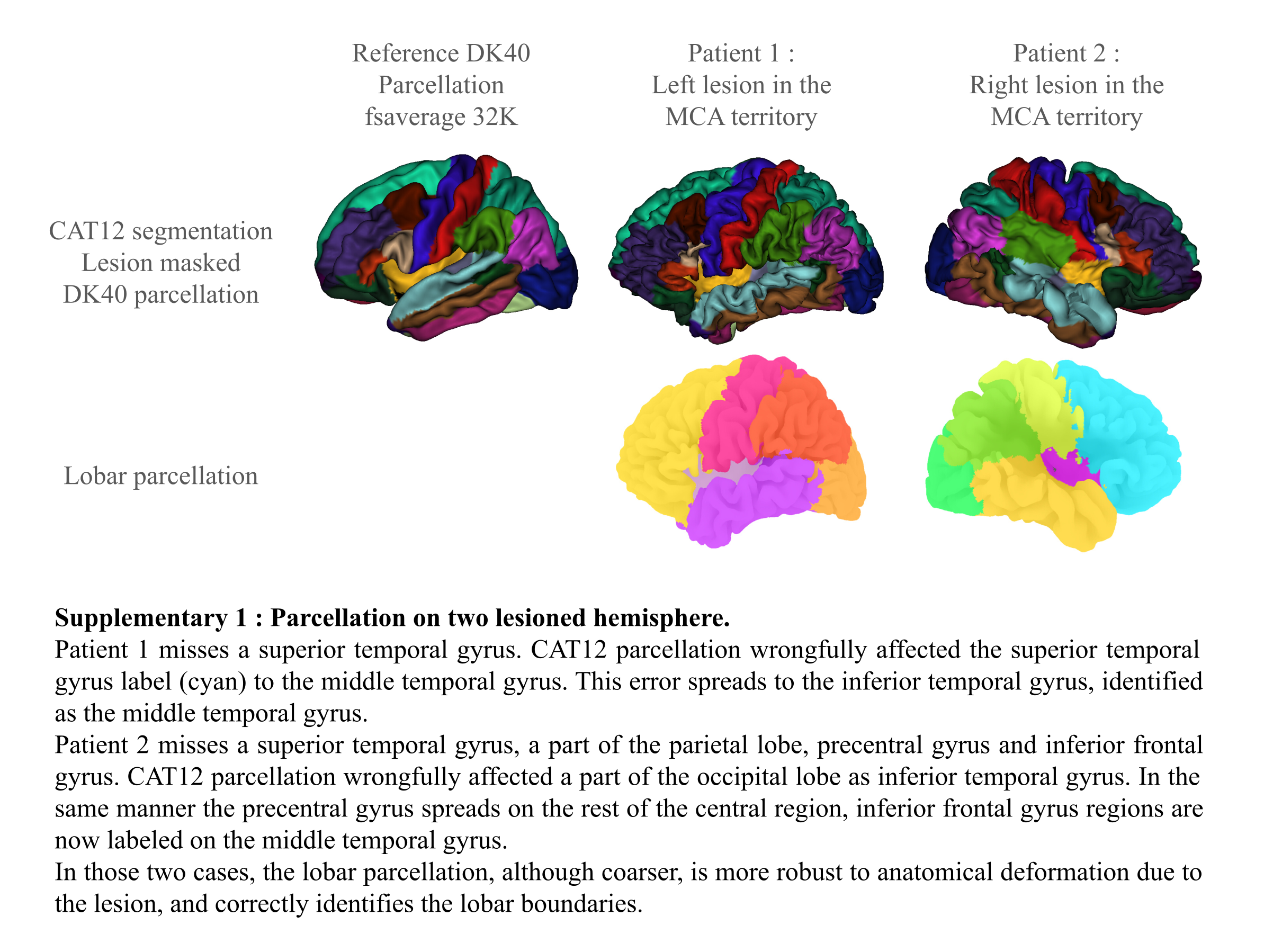

Supplement: Supplementary file 1 [file Image_1.TIF]

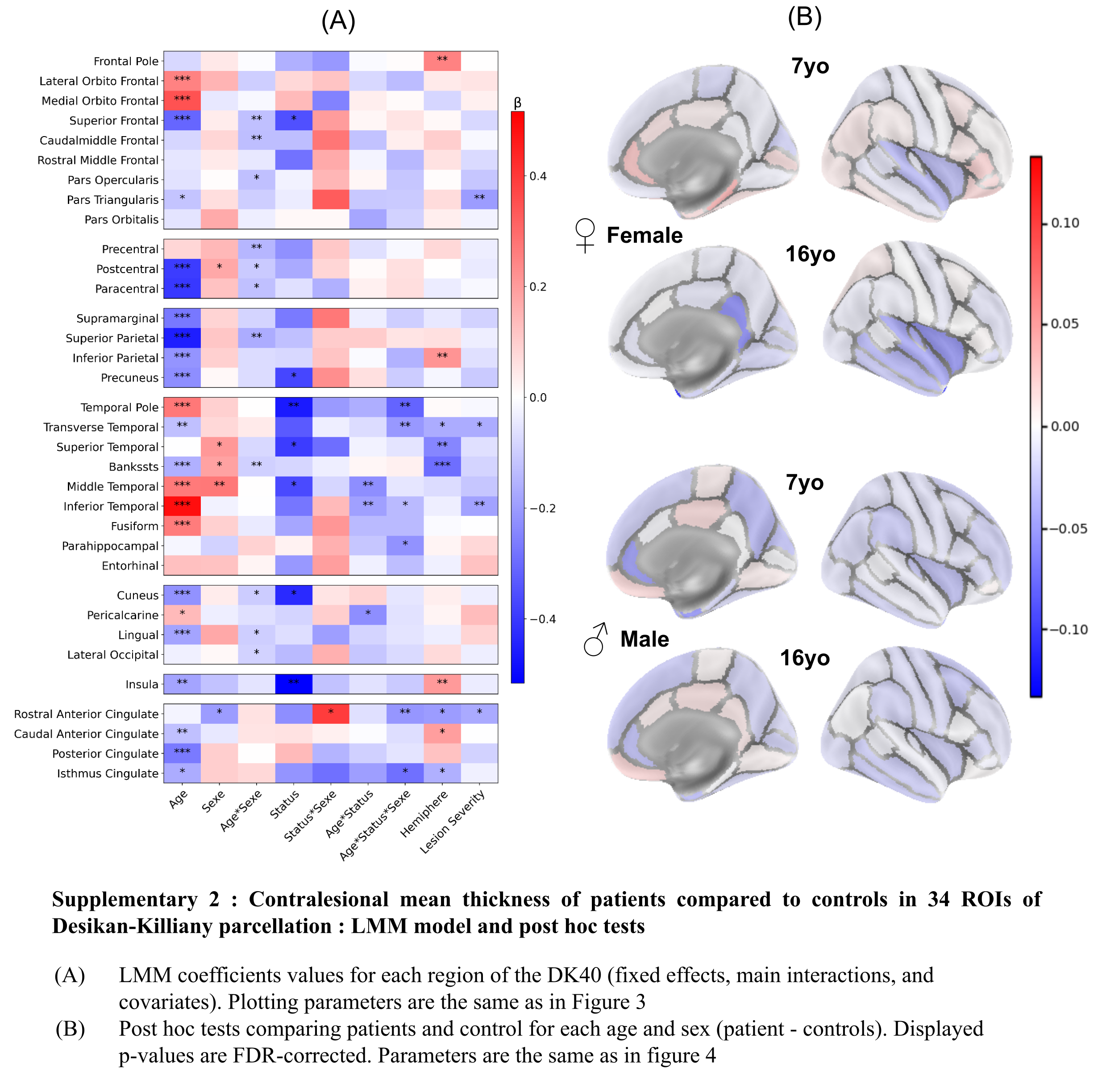

Supplement: Supplementary file 2 [file Image_2.TIF]

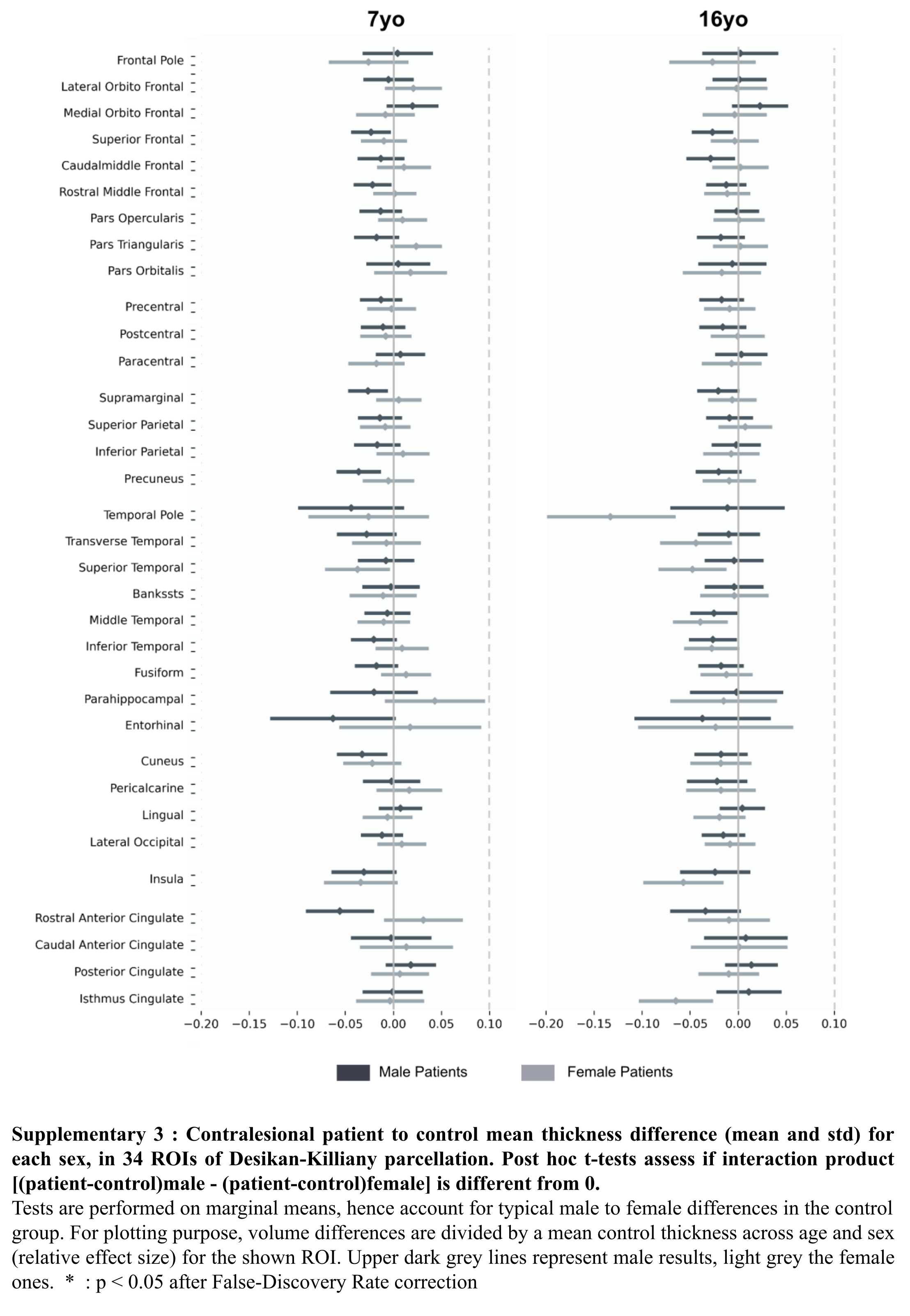

Supplement: Supplementary file 3 [file Image_3.TIF]

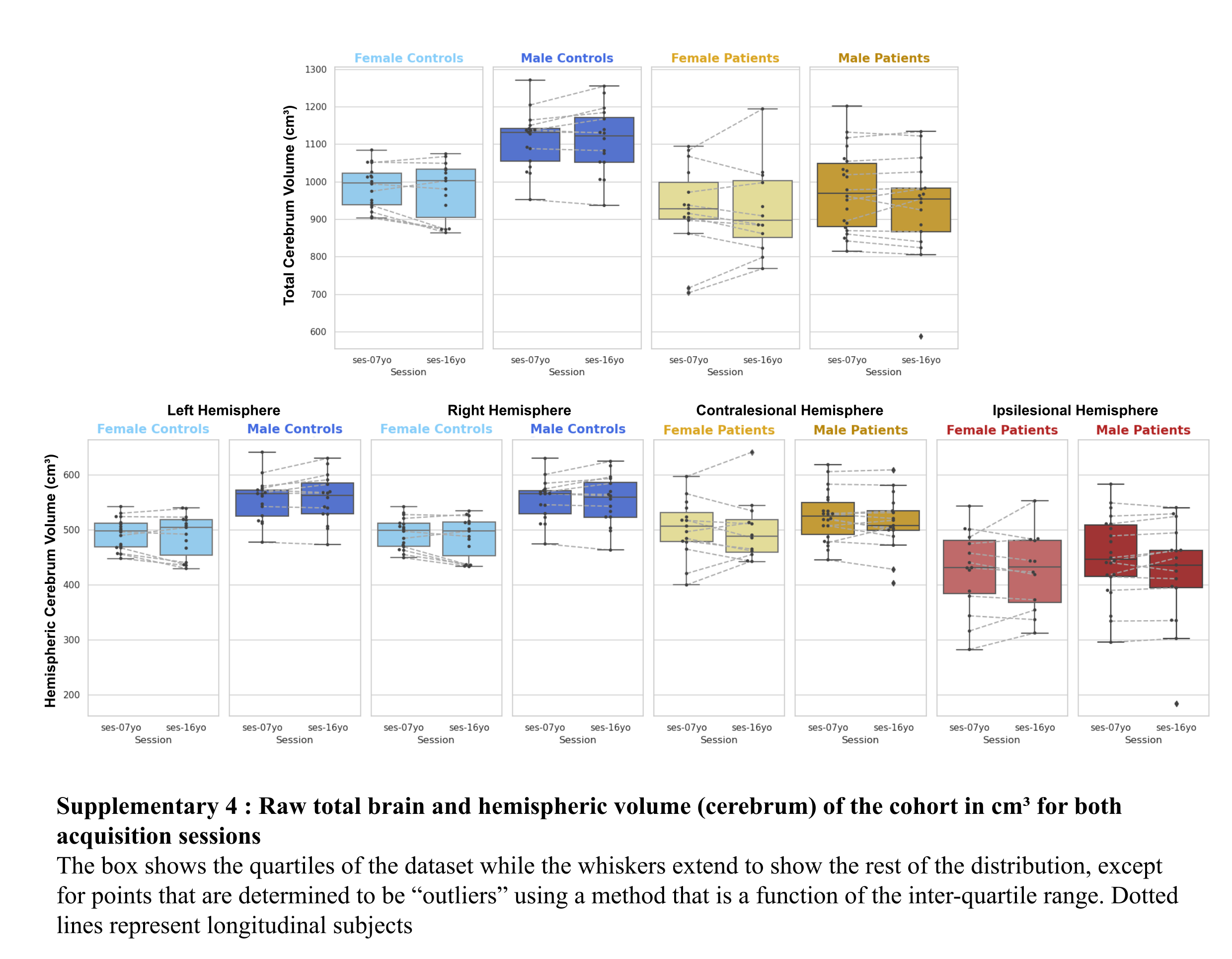

Supplement: Supplementary file 4 [file Image_4.tif]

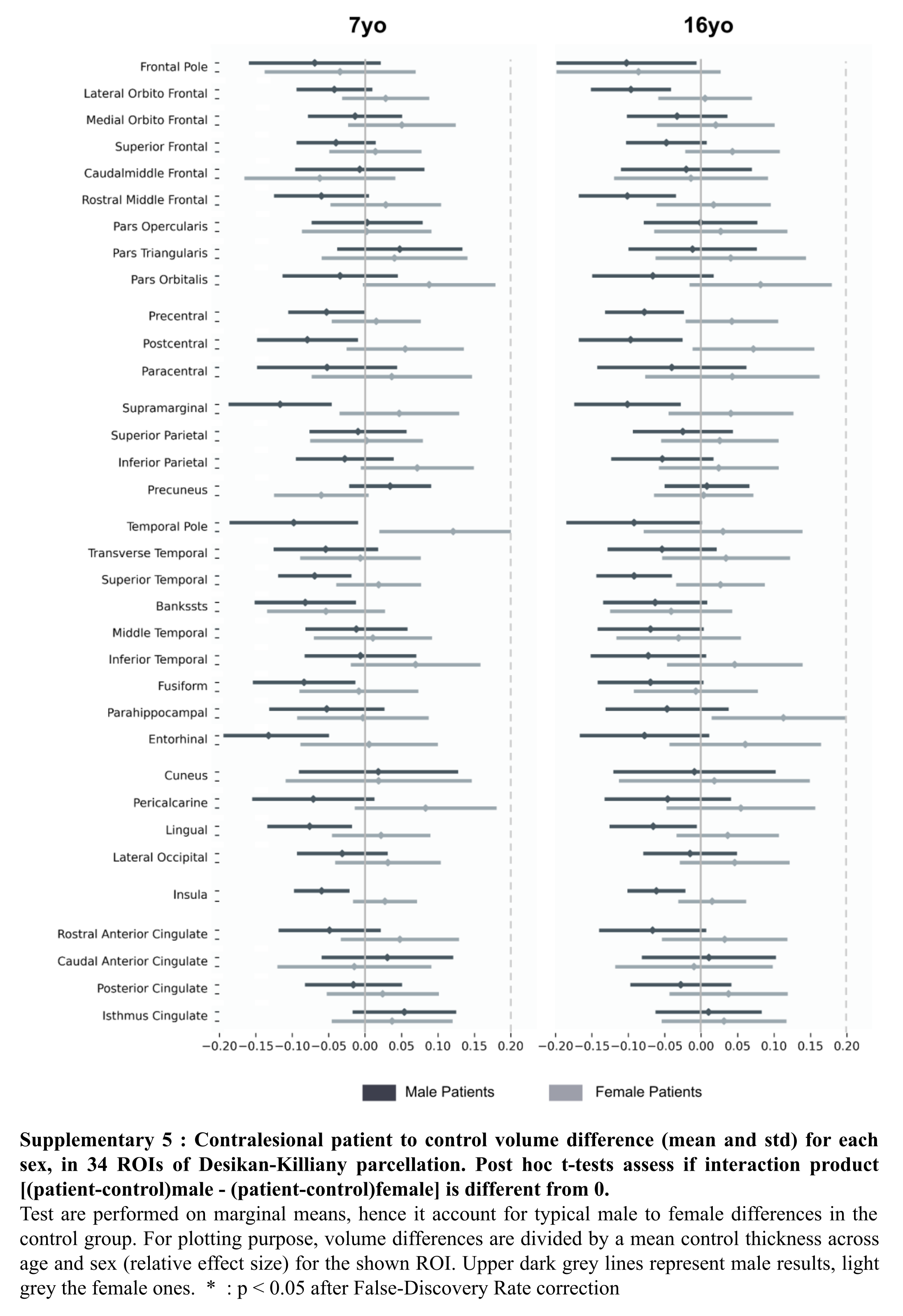

Supplement: Supplementary file 5 [file Image_5.TIF]

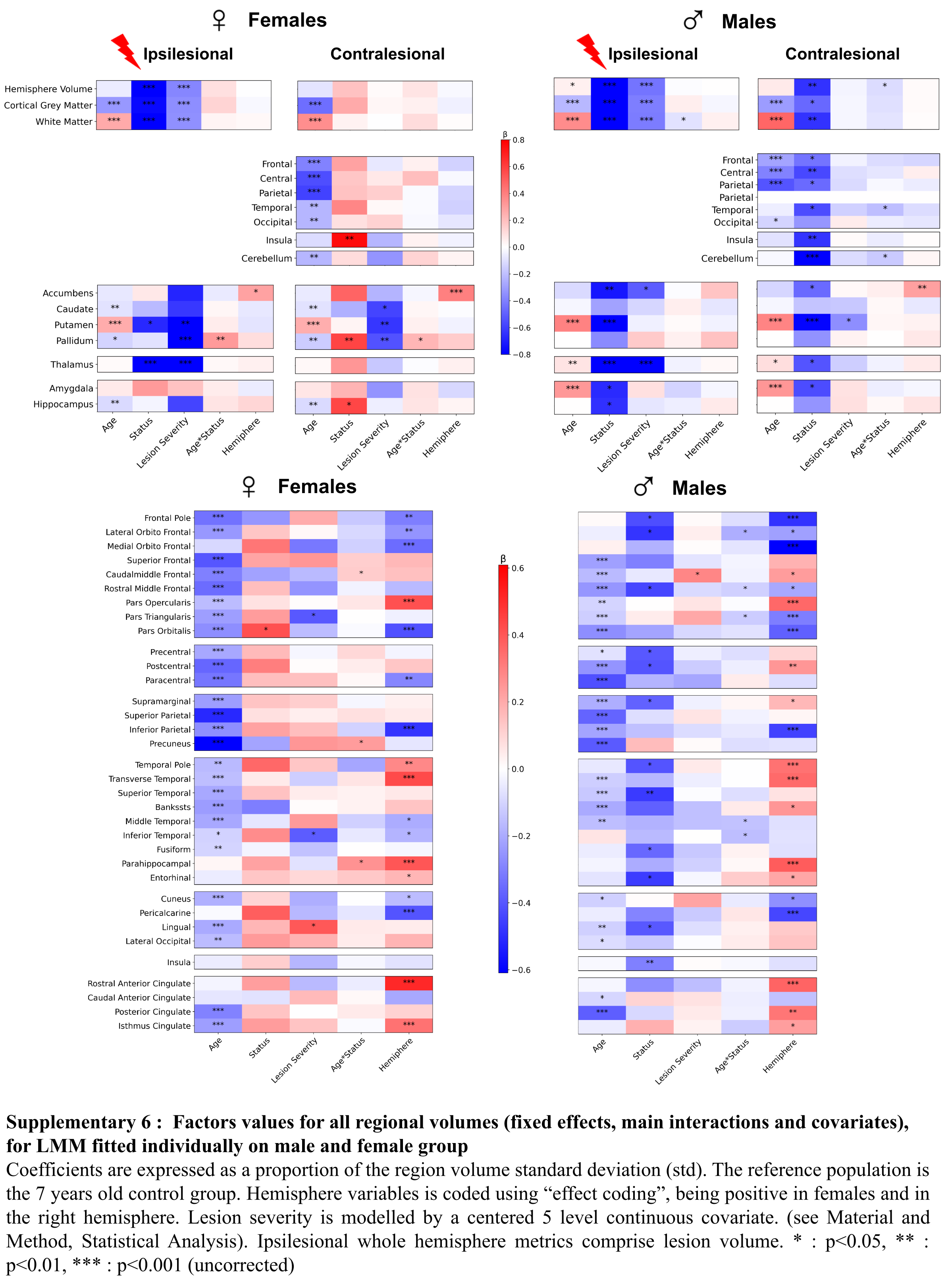

Supplement: Supplementary file 6 [file Image_6.TIF]

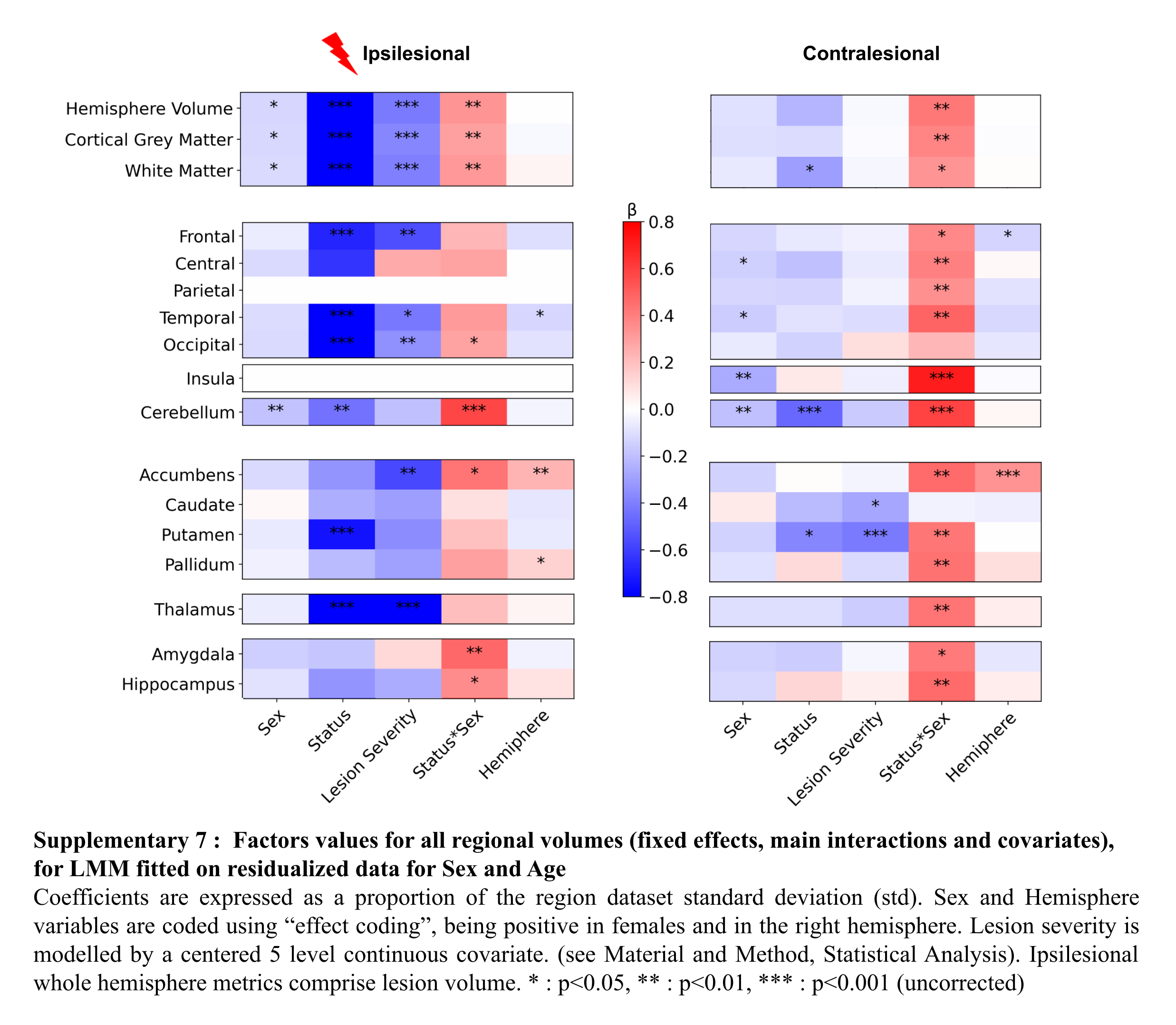

Supplement: Supplementary file 7 [file Image_7.TIF]

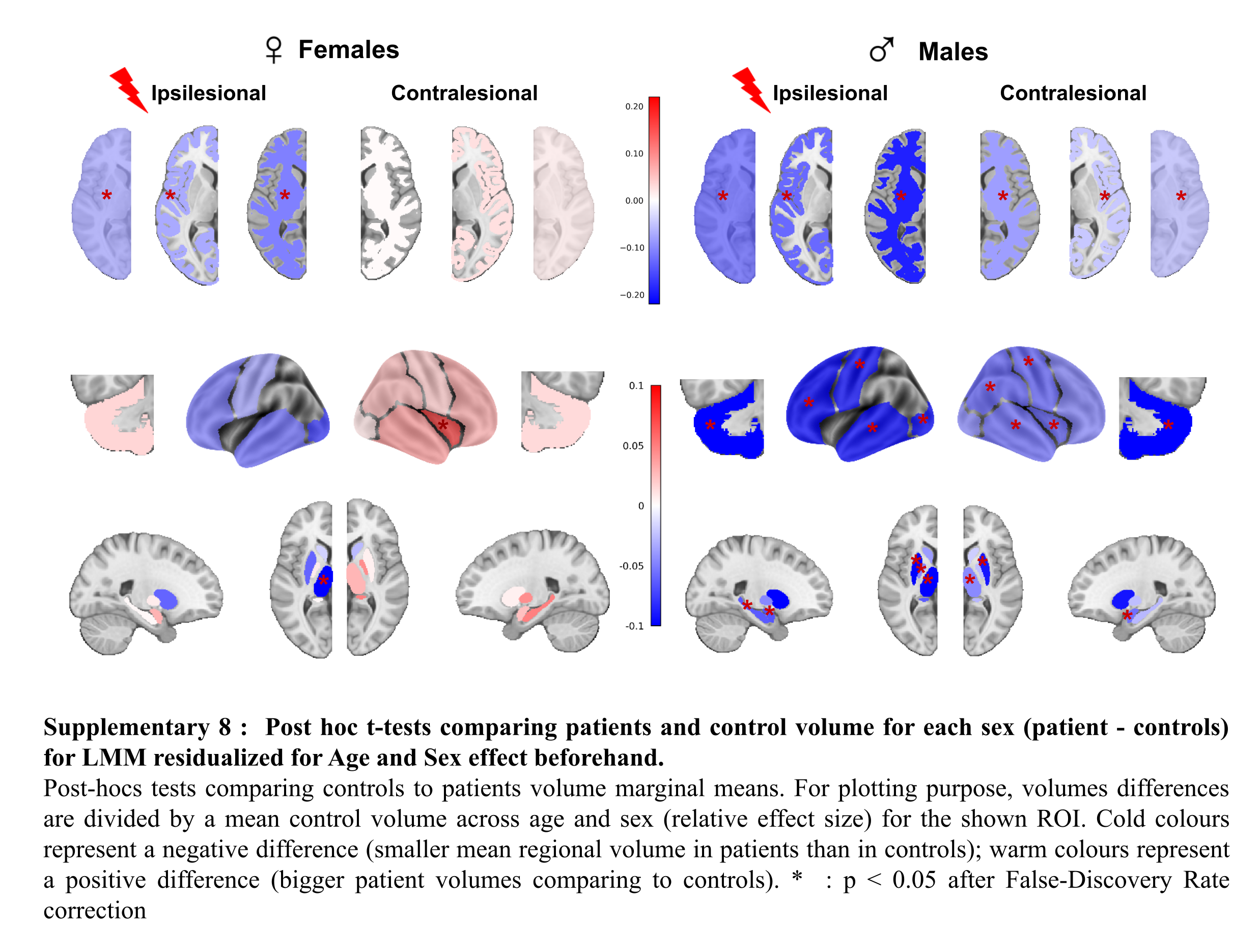

Supplement: Supplementary file 8 [file Image_8.TIF]

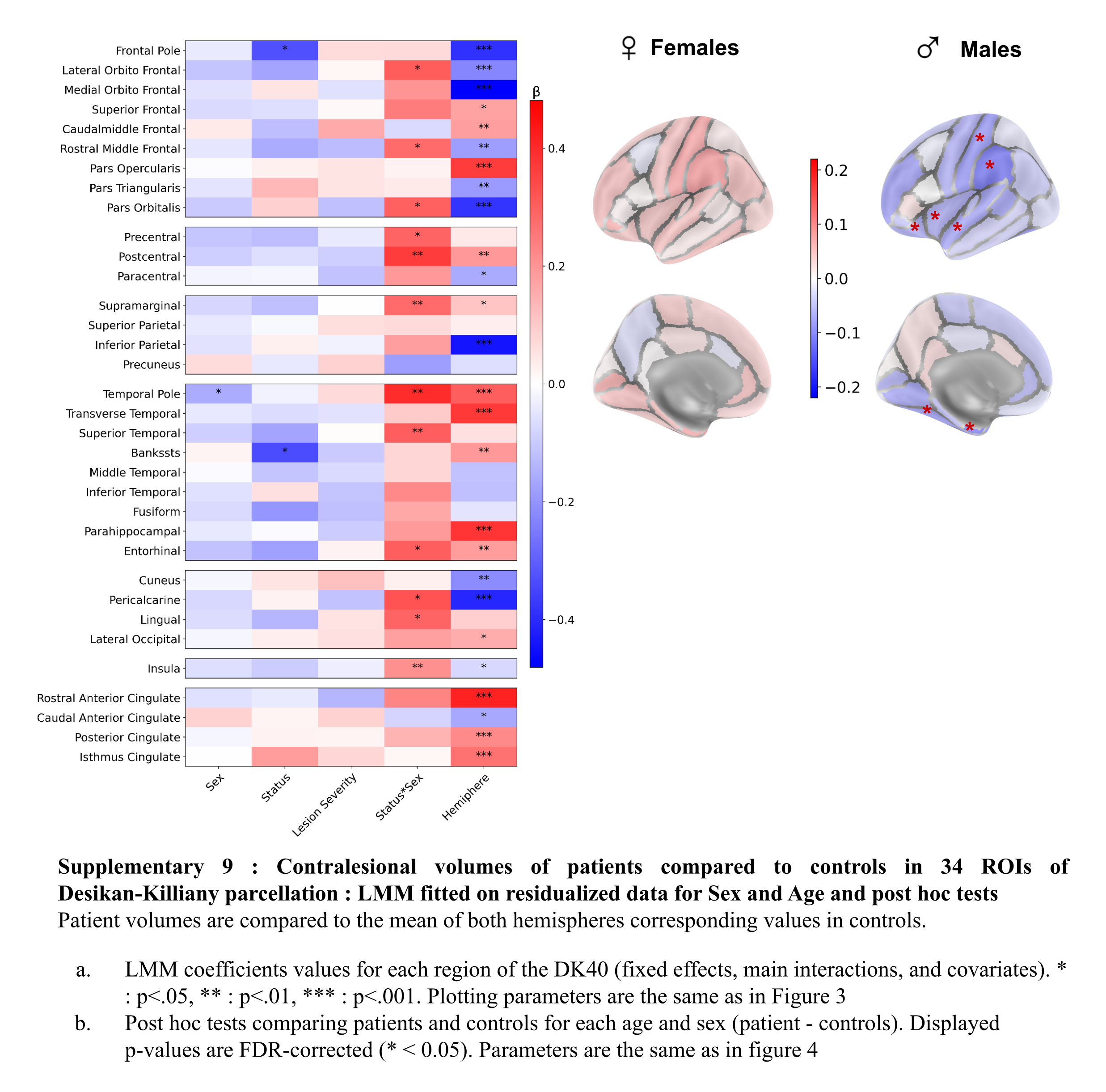

Supplement: Supplementary file 9 [file Image_9.TIF]

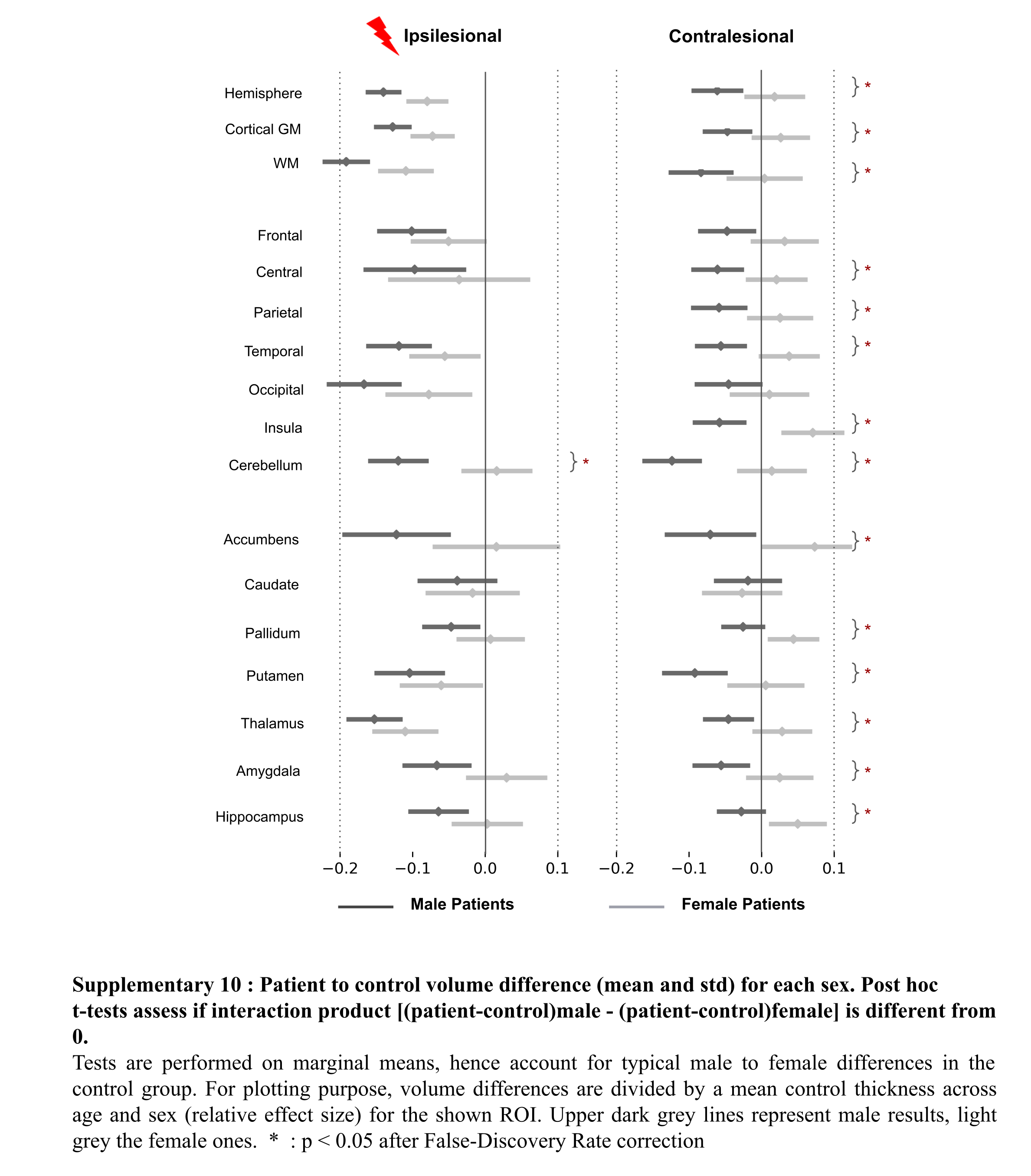

Supplement: Supplementary file 10 [file Image_10.TIF]

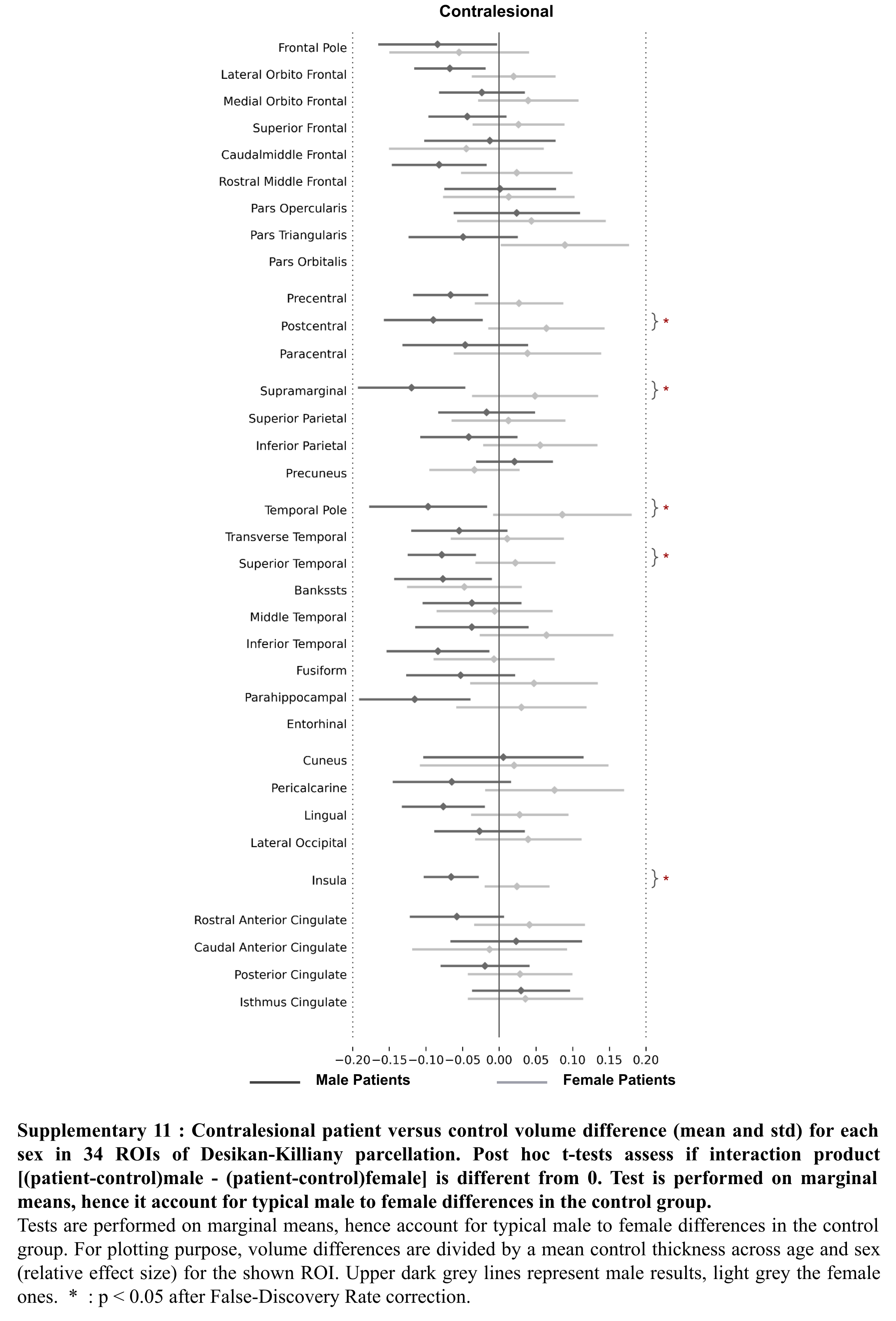

Supplement: Supplementary file 11 [file Image_11.TIF]
